# Proteins from formalin-fixed paraffin-embedded prostate cancer sections that predict the risk of metastatic disease

**DOI:** 10.1186/s12014-015-9096-3

**Published:** 2015-09-16

**Authors:** Jonathan C. Dunne, David S. Lamb, Brett Delahunt, Judith Murray, Peter Bethwaite, Peter Ferguson, John N. Nacey, Sven Sondhauss, T. William Jordan

**Affiliations:** Centre for Biodiscovery, School of Biological Sciences, Victoria University of Wellington, PO Box 600, Wellington, New Zealand; Prostate Cancer Trials Unit, Department of Pathology and Molecular Medicine, University of Otago Wellington, Wellington, New Zealand; Department of Surgery and Anaesthesia, University of Otago Wellington, Wellington, New Zealand

**Keywords:** Formalin-fixed paraffin embedded, Prognostic biomarkers, Prostate cancer, Proteomics

## Abstract

**Background:**

Prostate cancer is the most frequently diagnosed cancer in men and the third leading cause of cancer related deaths among men living in developed countries. Biomarkers that predict disease outcome at the time of initial diagnosis would substantially aid disease management.

**Results:**

Proteins extracted from formalin-fixed paraffin-embedded tissue were identified using nanoflow liquid chromatography-MALDI MS/MS or after separation by one- or two-dimensional electrophoresis. The proteomics data have been deposited to the ProteomeXchange with identifier PXD000963. A list of potential biomarker candidates, based on proposed associations with prostate cancer, was derived from the 320 identified proteins. Candidate biomarkers were then examined by multiplexed Western blotting of archival specimens from men with premetastatic disease and subsequent disease outcome data. Annexin A2 provided the best prediction of risk of metastatic disease (log-rank Chi squared p = 0. 025). A tumor/control tissue >2-fold relative abundance increase predicted early biochemical failure, while <2-fold change predicted late or no biochemical failure.

**Conclusions:**

This study confirms the potential for use of archival FFPE specimens in the search for prognostic biomarkers for prostate cancer and suggests that annexin A2 abundance in diagnostic biopsies is predictive for metastatic potential. Protein profiling each cancer may lead to an overall reduction in mortality from metastatic prostate cancer as well as reduced treatment associated morbidity.

**Electronic supplementary material:**

The online version of this article (doi:10.1186/s12014-015-9096-3) contains supplementary material, which is available to authorized users.

## Background

Prostate cancer (PCa) is the most frequently diagnosed cancer in men and the third leading cause of cancer related deaths among men living in developed countries [1]. Serum prostate-specific antigen (PSA) testing used to assist in making the diagnosis of PCa is a useful tool, but it has some limitations. It suffers from poor specificity, and elevation of the serum PSA is often due to non-malignant causes. The biopsies performed as a result of an elevated serum PSA result in the detection of some clinically irrelevant tumors (over-diagnosis) which will not trouble affected men during their lifetimes. Finally, the degree of elevation of the serum PSA level at the time of diagnosis does not assist in predicting how aggressively the cancer will behave.

Trial 96.01 was the first of two large randomized trials run by the Trans-Tasman Radiation Oncology Group (TROG) investigating new treatment strategies for locally advanced PCa. The trial end points at 10 years were reported in 2011 [2]. In trial patients, there appeared to be three distinct patterns of behaviour when the cancer relapsed: one with a short PSA doubling time of less than 4 months, one with an intermediate duration PSA doubling time of 4–9 months, and the other with a PSA doubling time greater than 9 months [3]. The polarization of biological behaviour on relapse suggests there are inherent differences between cancers responsible for the speed at which they grow and their biological behaviour. If this hypothesis is correct, then PCa could have a number of characteristic patterns of protein expression capable of informing the patient and clinician how the cancer is likely to behave. Identification of men at the time of diagnosis that are at high risk of developing biochemical relapse (PSA failure) is fundamental to improving the treatment of locally advanced PCa, and to reducing the over-treatment of men that currently occurs. Trial 96.01 showed that PSA failure was predominantly due to the development of metastatic disease [2], and risk of PSA failure can therefore be used as a surrogate for risk of metastatic disease.

We therefore investigated protein variation associated with risk of PSA failure using archival formalin-fixed paraffin-embedded (FFPE) tissue from men for whom disease outcome details was already known. A dataset of proteins was constructed using three proteomic strategies: (1) two-dimensional electrophoresis (2DE) followed by MALDI MS/MS of tryptic digests of protein spots; (2) Gel-MS/MS based on protein separation by one-dimensional SDS-polyacrylamide gel electrophoresis followed by protein identification by MALDI MS/MS of tryptic digests of multiple bands cut from the gels; (3) LC MS/MS carried out by reversed phase HPLC of tryptic digests followed by off-line MALDI MS/MS of the chromatography fractions. Potential prognostic proteins selected from the dataset were examined using Western blotting, and four putative prognostic markers were tested against proteins extracted from an independent set of archival sections from patients with known disease outcome. Currently we report a study of protein variation associated with disease outcome that has been carried out to identify candidates for analysis in a larger, independent, sample set. We anticipate that detection of prognostic proteins would be used principally to determine which cancers in older patients require treatment. A secondary objective is to determine which younger patients might benefit from intensification of their treatment at time of diagnosis. We expect that protein profiling of PCa biopsy specimens would be performed at the time of initial diagnosis of disease for prediction of the likely behavior of the cancer.

## Results and discussion

**Fig. 1:**
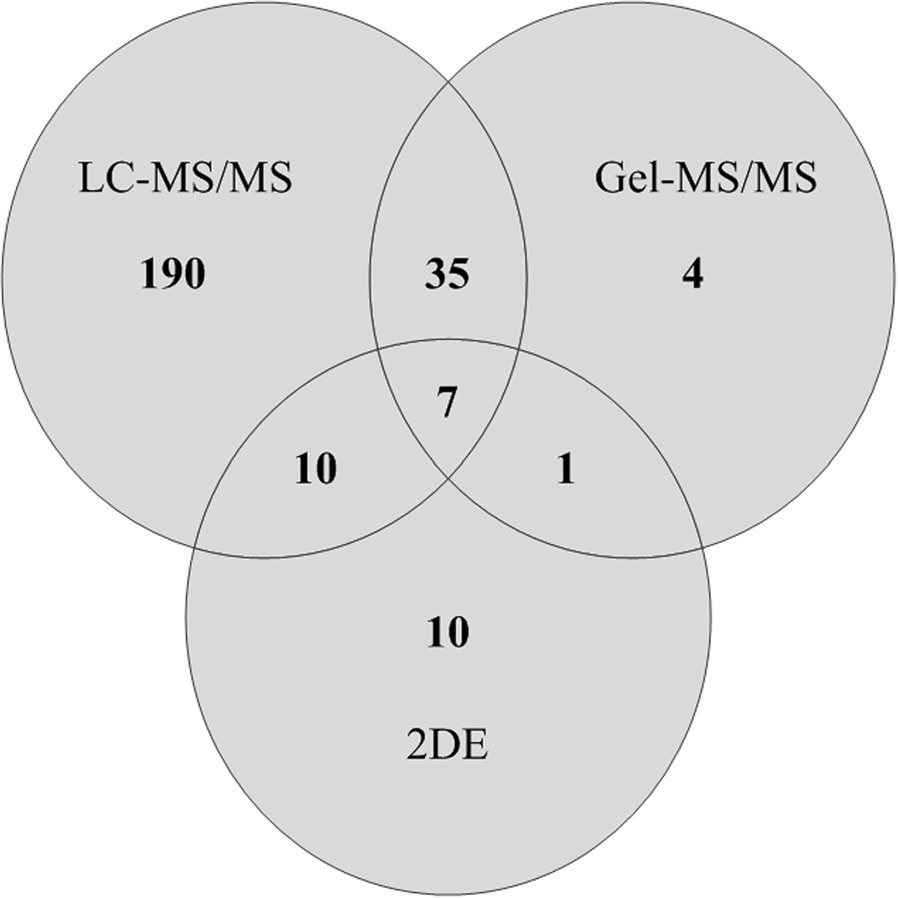
Summary of proteins identified by 2DE, Gel-MS/MS, and LC–MS/MS of radical prostatectomy sections. For Gel-MS/MS, total protein samples extracted from FFPE tissue were separated by 1DE and each lane was sliced horizontally into 30–35 slices. Proteins in each slice were trypsin digested and the resulting peptide mixtures analysed by MALDI TOF/TOF. For LC–MS/MS, total protein extracted from FFPE tissue was reduced, alkylated, and trypsin digested. Digests were then separated in triplicate by HPLC using a TEMPO LC-MALDI spotter system. Eluted peptides (16 s intervals) were mixed on-line with CHCA matrix and spotted for TOF/TOF analysis

Initially, a protein database was established using 2DE, Gel-MS/MS, and LC–MS/MS of proteins extracted from tumor regions of archival FFPE prostate tissue. Although 2DE separations gave relatively few discrete protein spots (Additional file 1) MALDI MS of tryptic digests of protein spots from these gels resulted in identification of 28 unique proteins including PSA (kallikrein-3). Western blotting with anti-PSA suggested multiple forms of this protein (Additional file 2), or of related kallikreins, but only the most abundant form was identified by MS. Gel-MS/MS identified 47 non-redundant proteins with at least one matched peptide, and using LC–MS/MS 242 non-redundant proteins were identified. Of the 242 proteins identified by LC–MS/MS, 190 were not detected by either of the other methods. Identity scores and % coverage for protein identification were often low, probably due to covalent modification of proteins during tissue processing [4]. In total, 320 non-redundant proteins were identified (Fig. 1, Additional files 3, 4, 5). The greatest numbers of identifications were of high abundance cytoplasmic, cytoskeletal and organelle matrix proteins including nuclear histones, ribosomal proteins and organelle Rabs that are detected during proteomic analysis of many mammalian tissues including prostate. Given the greater number of proteins identified by LC–MS/MS, strategies including LC–MS/MS of bands excised from SDS PAGE gels that enhance detection of proteins including integral membrane proteins should be investigated in future studies. For comparison, there are relatively few published analyses of FFPE prostate tissue since the initial publication of Hood et al. who used LC–MS/MS to examine tryptic digests of extracts from benign prostate hyperplasia and cancer regions of a FFPE prostate block [5]. Subsequent reports using frozen tissue include 2D-DIGE analysis of prostatectomy specimens from patients with or without relapse [6], and an iTRAQ comparison of tissue from localized and advanced prostate cancer patients [7].

**Fig. 2:**
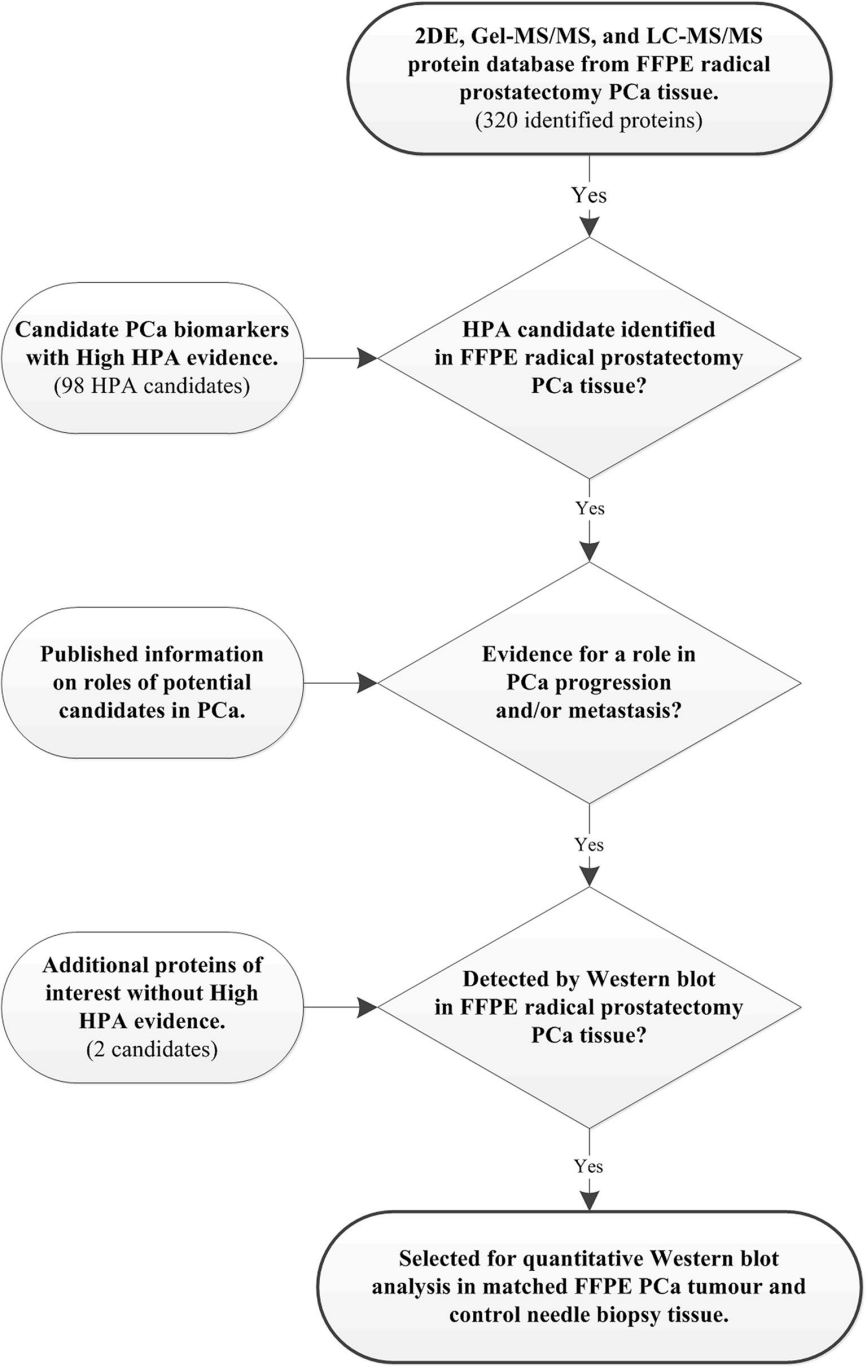
Strategy for selection of candidates for Western blot analysis. Potential candidates were initially selected from the set of 320 proteins identified by MS of radical prostatectomy sections by comparison with proteins annotated for PCa in the Human Protein Atlas (HPA, http://www.proteinatlas.org) and then further selected by literature searches for known associations with PCa (Additional file 6). A refined set of four candidates was examined by Western blotting of archival FFPE specimens for prediction of risk of metastatic disease

**Fig. 3:**
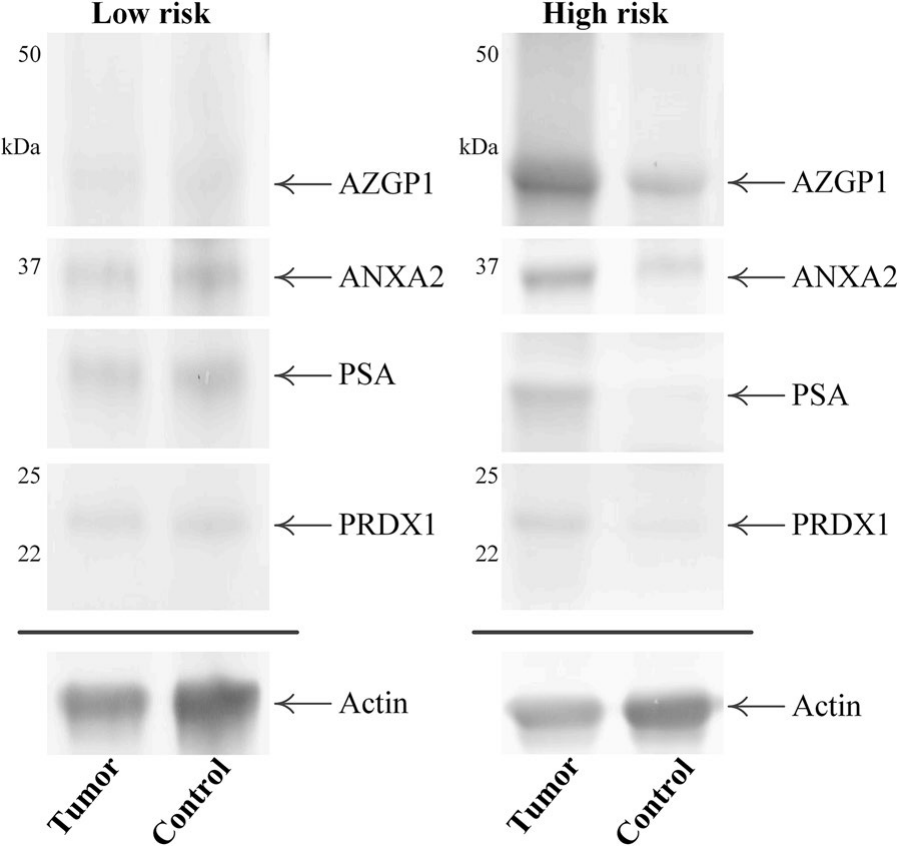
Representative Western blot images of matched tumor and control regions from patients with low or high risk of early biochemical failure. Proteins were separated by 1D-PAGE, transferred to Hybond-LFP membrane and probed for ANXA2, AZGP1, PSA and PRDX1, plus actin as a loading control. Detection with AlexaFluor-labelled second antibodies was captured using a FLA-5100 scanner

Selection of a set of potential prognostic biomarkers was carried out by interfacing the database of identified proteins with a search of the Human Protein Atlas (http://www.proteinatlas.org/ version 12) for candidates with known immunological reactivity with PCa (Fig. 2). Human Protein Atlas proteins corresponding to keyword “Prostate”; protein class “Candidate cancer biomarkers”; HPA evidence “High”, and filtered for IHC staining in PCa, when interfaced with the MS datasets gave a set of 12 proteins common to both lists (Additional file 6). Five of these candidates were selected on the basis of known associations with PCa (see below) for initial examination by Western blotting of FFPE extracts of radical prostatectomy tissue but only four were detected at sufficient abundance for subsequent analysis. This included PSA and three other proteins that have been associated with PCa: annexin A2 (ANXA2) [8–11], zinc-alpha-2-glycoprotein (AZGP1, ZAG) [12–14] and peroxiredoxin-1 (PRDX1) [15, 16]. Positive reactions at the expected molecular masses were obtained for each of these proteins, and after optimising primary and secondary antibody dilutions a multiplexing strategy was developed by blotting with a mixture of antibodies to all four proteins plus actin as a loading control (Fig. 3). The multiplex strategy allowed detection of several proteins from a single blot and facilitated analysis of the small amount of tissue that was harvested from some FFPE blocks. Heat shock protein beta-1 was also detected by Western blotting but often with low intensity signals near the threshold for detection and was not included in the statistical analysis.

Tumor and control tissue regions excised from FFPE sections from 16 patients, independent of tissue used for the prior MS analyses, were examined using a multiplexing method. The samples for multiplexed Western blotting were chosen to represent a range of disease outcomes (time to biochemical failure) with five cases >100 months, five cases ≤40 months, and six “intermediate” 50–87 months. Measured protein abundances were normalized to actin amounts and the tumor/control abundance ratio for each protein was used for analysis of association with time to biochemical failure. The ratios of tumor to control tissue values were used to correct for possible inter-individual differences in protein abundance not related to PCa. In most cases the amount of tissue available from the 16 cases was only sufficient for a single extraction and Western blot. Following detection of the associations of ANXA2 and PSA with disease outcome (see below), analytical reproducibility for the two proteins was examined by replicate analyses of proteins extracted from FFPE radical prostatectomy tissue. Four separate protein extractions were carried out from a radical prostatectomy block and the set of four extracts was each analyzed twice by Western blotting using 15 μg of protein on each gel. CVs for the normalized, relative abundance values (ANXA2 or PSA normalized against the actin loading control) of the four extracts ranged from 4.4 to 9.5 % for ANXA2 and 2.1–6.5 % for PSA, with less than 6.5 % variation for either protein between the duplicate Western blots.

**Fig. 4:**
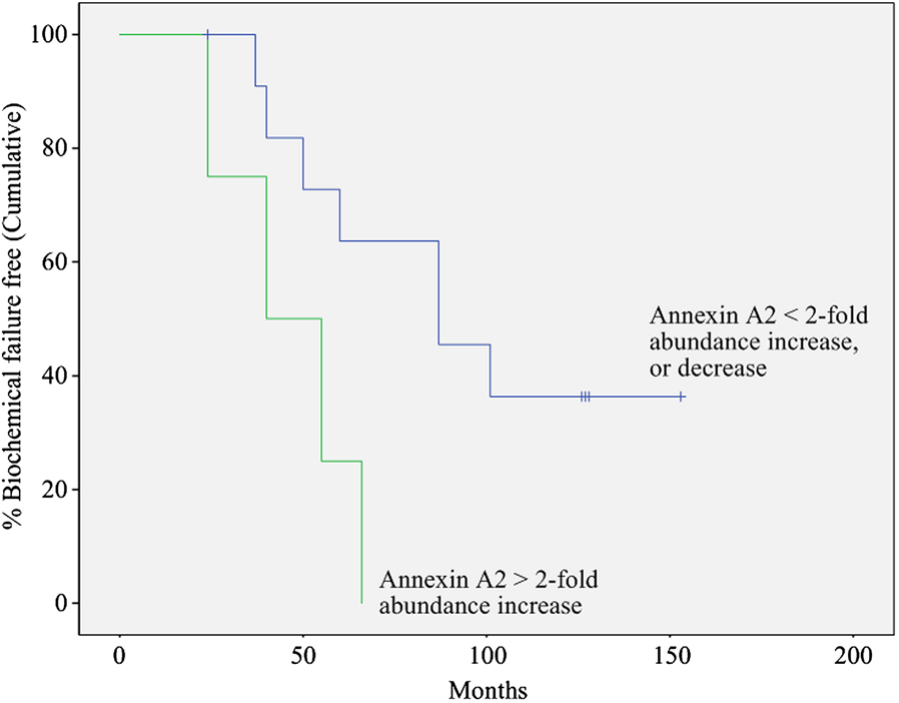
Biochemical failure free survival as a function of Annexin A2 risk stratification group. Kaplan–Meier estimator curves of time to biochemical failure were constructed using an a priori selected two-fold increase in ANXA2 abundance in tumor relative to control tissue as the cut-point in each of the 16 cases. Statistical analysis of the failure demonstrated a significant difference in time to biochemical failure between the two groups (log-rank Chi squared p = 0. 025), indicating that a >2-fold change in ANXA2 abundance is predictive for early biochemical failure. Vertical lines on the Annexin A2 <2 curve denote censored cases

For each protein of interest in the 16 cases the Kaplan–Meier estimator curves of the “survival” (biochemical failure) function were stratified using cut points determined before the analysis and the failure curves compared using the log-rank Chi squared test. For two of the proteins there was a significant difference in the failure curves between the strata predefined by differences in protein abundance measurements. The Kaplan–Meier curves for ANXA2 (Fig. 4) demonstrate that for men with greater than a two-fold increase in ANXA2 abundance in tumor relative to control tissue this appears to predict those men who will experience early biochemical failure during the trial period (log-rank Chi squared p = 0. 025). All such men experienced biochemical failure within 66 months. In contrast, 60 % of the men who exhibited less than a two-fold increase, or decrease, in ANXA2 abundance at the time of diagnosis were biochemical failure free after 66 months, and more than 35 % of them remained biochemical failure free at the trial end-point. The Kaplan–Meier curves for PSA using a three-fold cut-off for protein abundance in tumor relative to control tissue also demonstrated a significant difference in failure risk (log-rank Chi squared p = 0. 016) (Additional file 7). All men with greater than a three-fold increase in PSA abundance experienced biochemical failure within 87 months, whereas more than 55 % of those with less than a three-fold increase remained biochemical failure free at the trial end-point.

Multivariate analysis, using Cox proportional hazard modeling was applied to explore the relationship between the relative abundance change of each protein, modeled as continuous variables, and time to biochemical failure at the same time controlling for any difference in treatment employed (Table 1). Despite the small size of the dataset, and after controlling for all other protein relative abundance changes, the relative abundance change of ANXA2 in tumor versus control tissue was a significant and independent predictor of the risk of biochemical failure (p = 0.018). The apparent association with PSA expression seen in the univariate analysis was not supported in the multivariate modeling.

**Table 1:** Cox proportional hazard modeling of target protein abundance and time to biochemical failure

		Number	Percent
Cases available in analysis	Event^a^	11	68.8
	Censored^b^	5	31.2
	Total	16	100.0

Variables in the equation	Regression coefficient	Standard error	*p* value
ANXA2	0.427	0.181	0.018
AZGP1	−0.038	0.035	0.276
PRDX1	−0.165	0.108	0.127
PSA	0.135	0.101	0.181
Treatment arm^c^	0.607	0.918	0.509

^a^Dependent variable: time to biochemical failure (months)

^b^Biochemical failure did not occur within the trial period

^c^Radiotherapy plus either 3 or 6 months androgen deprivation therapy (Additional file 8)

ANXA2 is a 36 kDa peripheral membrane protein belonging to the 12 member annexin family of calcium binding proteins. ANXA2 is found on endothelial cells, early myeloid cells, and a variety of tumor cell types [17–20], and has been demonstrated to mediate cellular processes that are essential for cancer metastasis such as tumor cell migration [21, 22], invasion [23, 24] and adhesion [25]. Increased expression of ANXA2 has been reported in tumors of the breast, liver, and pancreas [23, 26, 27]. There is debate surrounding the changes observed in ANXA2 abundance with different types of PCa. The expression of ANXA2 is reduced in high-grade prostatic intraepithelial neoplasia and in low- to moderate-grade PCa [28], but becomes elevated in high-grade PCa [10, 29, 30]. Of interest also is the observation of Hood et al. [5] that ANXA2 apparently did not differ in abundance between cancer and benign hyperplasia regions of an FFPE block, although their analysis differs of course from our study of protein change that predicts disease outcome. Shiozawa et al. demonstrated recently that ANXA2 and its receptor are involved in PCa metastasis by regulating tumor cell migration and adhesion to osteoblasts and endothelial cells, as well as tumor cell proliferation at metastatic sites [30]. In contrast, Hailermarian et al. [31] did not find a statistically significant association of ANXA2 or three other immunohistochemical markers with risk of PCa. Our demonstration that ANXA2 abundance in tumor versus control tissue at the time of diagnosis of localized, advanced PCa was significantly associated with disease progression therefore complements the immunohistochemical analyses of sections from PCa and high grade prostatic intraepithelial neoplasia.

In summary, in this study we analyzed archival FFPE tissue from men with locally advanced PCa for whom there was ten-year outcome data for disease progression. In general, information about protein modification and abundance obtained by Western blotting complements histological description of the cellular localization of proteins. Our strategy was to use proteomic techniques to compile a database of abundant proteins for selection of candidates for subsequent multiplex Western blot analysis. Pauly et al. [32] recently reported multiplexed antibody microarray analysis of proteins extracted from FFPE tissue. To our knowledge we describe the first application of multiplexed Western blotting for analysis of FFPE extracts from prostate biopsies. Our results complement those of Geisler et al. who used 2DE of frozen prostate specimens to show differences among proteins that included ANXA4 and A5, although they did not identify ANXA2, between patients with or without biochemical relapse [6].

Looking forward, use of ELISA or focused mass spectrometry [33, 34] for measurement of individual or panels of proteins from biopsy tissue may be used both for validation of prospective biomarkers and subsequently as diagnostic assays to enhance management of prostate cancer. There may be additional potential to develop urine or blood-based assays, although for proteins including ANXA2 that are also elevated in other cancers [35] measurements on prostate biopsies would be needed for specific diagnosis.

## Conclusions

In conclusion, this pilot proteomics study suggests that tumor expression of ANXA2 in diagnostic samples of a PCa may be predictive for the metastatic potential of that cancer. This study also suggests that PSA may have predictive potential. A larger study is in progress to examine and extend these findings using FFPE specimens from the Trans-Tasman TROG 03.04 trial [36] that is subsequent to the 96.01 trial used in the current study.

## Methods

### Study design and sample processing

FFPE tissue blocks containing prostate biopsies from Wellington men who entered the TROG 96.01 clinical trial were used in the current study. Proteomic studies on the biopsies were approved by the Wellington Ethics Committee. The biopsies were collected from 1996 to 2000 at the time of initial diagnosis before treatment and were immediately processed to FFPE blocks. The study design for TROG 96.01 has been described [2]. Eight hundred and eighteen men (ages 41–87), from Australia and New Zealand, who had locally advanced PCa without distant metastases, received either radiotherapy alone or radiotherapy plus androgen deprivation for 3 or 6 months. Benefits of androgen deprivation included better control of serum PSA (biochemical control) and PCa specific survival. For the current proteomic study, tissue was included from 16 men who were treated with radiation plus androgen deprivation [2]. For each man, the presenting tumor characteristics are provided in Additional file 8. The clinical endpoints were whether or not biochemical failure occurred, and if it did, the time from randomization to the event.

Initially, sections (20 µm thick, approximately 25 mm^2^) cut from a FFPE radical prostatectomy tissue block were used for investigation and optimization of protein extraction, 1DE, 2DE, MS and Western blotting. Protein recovery after incubation of 20 µm thick sections (approximately 25 mm^2^) in Extraction Buffer (see below) was 142 ± 58 µg protein/mg dry tissue (n = 2). Subsequently, Western blot analysis was carried out on proteins extracted from tumor and control regions of FFPE needle biopsies for statistical analysis of protein association with disease outcome. Tissue harvesting for proteomics was carried out by pathologists (BD and PF). FFPE sections (5 µm) from each block were stained with hematoxylin/eosin and examined to localize the site of the tumor. Tumor and control regions were excised from adjacent unstained sections under a dissecting microscope. FFPE sections (10 µm) were deparaffinised at room temperature using 1 mL xylene (3× 2 min), followed by rehydration in ethanol series (100 %, 90 %, 80 %, 70 %, 5 min each) and air dried.

Deparaffinised, air-dried tissue samples were incubated in 250 µL of Extraction Buffer consisting of 40 mM Tris–HCl (pH 8.2), 2 % SDS, and 3 % DTT (dithiothreitol) for 1 h at room temperature followed by 20 min at 100 °C [37]. Samples were immediately centrifuged at 14,000×*g* for 15 min and each supernatant was added to 1.25 mL of Precipitation Solution (ProteoExtract^®^ Protein Precipitation Kit, EMD Millipore, Billerica, MA) and incubated overnight at −20 °C before centrifugation at 14,000×*g* for 30 min at 4 °C. The protein pellets were washed 2× 5 min using 500 µL ProteoExtract^®^ Wash Solution, air-dried for 5 min and resuspended in either rehydration buffer (7 M urea, 2 M thiourea, 2 % CHAPS, 20 mM DTT, 0.5 % immobilized pH gradient buffer, trace bromophenol blue) for 2DE; 1× LDS sample buffer containing Sample Reducing Agent (Invitrogen, Carlsbad, CA) for Gel-MS/MS; or 50 mM Tris–HCl (pH 8.5), 8 M urea, 0.1 % SDS for LC–MS/MS. The protein concentration of each sample was assayed using a 2-D Quant Kit (GE Healthcare, Uppsala, Sweden). 1DE, 2DE, sample processing for mass spectrometry including digestion with sequencing-grade modified trypsin (Roche Applied Science, Penzberg, Germany), identification of proteins from 2DE gels by MALDI mass fingerprinting, and immunoblotting were carried out as previously described [38].

### Gel-MS/MS

Protein extracts (30 µg) from FFPE tissue blocks were separated in triplicate by 1DE on 8 × 8 cm 4–12 % NuPAGE gels and fixed for 90 min at room temperature in 50 % ethanol:3 % orthophosphoric acid, then washed 3× with water. Each sample lane was sliced horizontally into 30–35 slices that were then transferred to the wells of a 96-well microtiter plate and processed in a GE Healthcare Ettan digester. Each gel piece was diced into approximately 1 mm^3^ cubes, destained using 50 % methanol:50 mM NH_4_HCO_3_ (3× 45 min), and air-dried for 2 h. Proteins were digested for 6 h using 10 µL trypsin solution (2.5 ng/µL trypsin in 20 mM NH_4_HCO_3_) per well. Digest peptides were extracted using ACN (acetonitrile):0.1 % TFA (trifluoroacetic acid) 1:1 (3× 45 min), air-dried, then resuspended in 2 µL 10 mg mL^−1^ CHCA (alpha-cyano-4-hydroxycinnamic acid) in ACN:0.1 % TFA 1:1 and spotted onto an ABSCIEX 5800 MALDI TOF/TOF target plate.

### LC–MS/MS

Total protein extracts (50 µg) were reconstituted overnight at 4 °C in 50 µL of 50 mM Tris–HCl (pH 8. 5), 8 M urea, 0.1 % SDS. Proteins were reduced in 5 mM DTT for 1 h, alkylated for 30 min in 10 mM iodoacetamide, and quenched in 15 mM DTT, all at room temperature. Samples were diluted 10-fold by the addition of 450 µL of 50 mM Tris–HCl (pH 8.5) and digested overnight at room temperature using 2.5 µg of trypsin per sample. Digests were lyophilised, resuspended in 500 µL 0.1 % TFA and purified using Omix C18 reverse-phase 100 µL tips (Agilent Technologies, Santa Clara, CA). Peptides were eluted into 100 µL of ACN:0.1 % TFA 7:3, lyophilised, and resuspended in 30 µL 0.1 % TFA. Reverse-phase LC-MALDI was performed in triplicate using a TEMPO LC-MALDI spotter system (AB SCIEX, Framingham, MA) with a 150 × 0.1 mm Chromolith^®^ CapRod^®^ RP-18e monolithic column (Merck Millipore, Billerica, MA). Five microlitres of each sample was injected into a 3 µL sample loop at 1 µL min^−1^. Peptide separation was achieved using a mobile phase system comprised of 2 % ACN, 0.1 % TFA (Reagent A) and 98 % ACN-0.1 %TFA (Reagent B) with a 36 min linear gradient of 0–80 % Reagent B at 1 µL min^−1^. Eluted peptides were mixed on-line with MALDI matrix (CHCA in ACN-0.1 % TFA 1:1 at 1 µL min^−1^) and spotted at 16 s intervals onto an ABSCIEX 5800 target plate.

### Mass spectrometry and database searches

Gel-MS/MS and LC–MS/MS spectra were collected using an ABSCIEX 5800 MALDI TOF/TOF mass spectrometer in positive ion mode. TOF/TOF data files were searched against UniProtKB human sequences (88,473 sequences, final searches 17 January 2014) using ProteinPilot v3.0 (AB SCIEX) with the Paragon algorithm [39]. Search parameters were maximum one missed trypsin cleavage, maximum 50 ppm and 0.2 Da mass tolerances for MS and MS/MS spectra respectively, cysteine carbamidomethylation as a fixed modification, and methionine oxidation as a variable modification. Paragon searches were conducted in “Thorough Mode” using a reversed sequence database to obtain 95 % peptide identification confidence. The mass spectrometry proteomics data have been deposited to the ProteomeXchange Consortium (http://www.proteomexchange.org) via the PRIDE partner repository [40] with the dataset identifier PXD000963 and doi 10.6019/PXD000963.

### Western blotting

Western blot analysis was carried out on proteins extracted from tumor and control regions of FFPE needle biopsies for statistical analysis of protein association with disease outcome. Protein extracts (15–20 µg protein) were resolved by 4–12 % 1D-PAGE and transferred to Hybond-LFP membranes (GE Healthcare) for 1 h at 30 V. The membranes were blocked in PBS containing 0.1 % Tween-20 and 0.25 % gelatin for 90 min at room temperature and then probed overnight at room temperature with the following antibodies: ANXA2 (C-10, mouse monoclonal, Santa Cruz, sc-28385) 1:100; PSA (C-19, goat polyclonal, Santa Cruz, sc-7638) 1:200; PRDX1 (N-19, goat polyclonal, Santa Cruz, sc-7381) 1:200; AZGP1 (H123, rabbit polyclonal, Santa Cruz, sc-11358) 1:200. Membranes were then incubated sequentially for 1 h at room temperature with the following fluorophore conjugated secondary antibodies: AlexaFluor^®^647 goat anti-rabbit IgG (H+L), AlexaFluor^®^555 goat anti-mouse IgG (H+L), and AlexaFluor^®^647 chicken anti-goat IgG (H+L), all 1:2500. Images were acquired immediately after incubation with each secondary antibody using a FLA-5100 fluorescent scanner (FujiFilm, Tokyo, Japan), and signal intensity was quantified using ImageJ™ software [41]. Each membrane was also probed for 3 h at room temperature with anti-actin primary antibody (mouse monoclonal, Chemicon, MAB1501) followed by AlexaFluor^®^555 goat anti-mouse IgG (H+L) 1:2500. The intensity of each band was measured in triplicate to establish a mean signal intensity and standard deviation for each protein including the actin loading control. The mean values for each protein were normalized to the relevant actin control values and relative abundance changes for each protein were calculated as the ratio of normalized tumor to control tissue signal intensity. Protein abundance analysis was performed without knowledge of disease outcome for each of the clinical samples.

### Statistical methods

SPSS software was used for statistical analysis (IBM SPSS Statistics 22.0—August 2013). The association of protein abundance measurements with time to biochemical failure was assessed in a univariate way using the Kaplan–Meier estimator for the survival function with cut-points chosen a priori while multivariate associations with biochemical failure were assessed by application of Cox proportional hazard modeling.

Western blot signals were undetectable in 9.4 % of the 160 individual protein relative abundance measurements (four target proteins plus actin measured in tumor and control tissue for each the 16 cases). Missing values were imputed with a value that was approximately 50 % of the lowest detectable value for any protein in either tissue type.

## Additional files

**Additional file 1:** Two-dimensional electrophoresis of proteins extracted from FFPE tissue.
**Additional file 2:** Two-dimensional Western blot analysis of PSA.
**Additional file 3:** Summary of proteins identified by MALDI MS/MS of spots excised from 2DE gels.
**Additional file 4:** Summary of proteins identified in FFPE prostate tumor extracts using Gel-MS/MS.
**Additional file 5:** Summary of proteins identified in FFPE prostate tumor extracts using LC-MS/MS.
**Additional file 6:** Summary of the proteins identified in FFPE PCa tissue that were selected for Western blot (WB) analysis.
**Additional file 7:** Biochemical failure free survival as a function of PSA risk stratification group.
**Additional file 8:** Summary of patient samples analysed.

## Abbreviations

1DE: one-dimensional electrophoresis
2DE: two-dimensional electrophoresis
ACN: acetonitrile
ANXA2: annexin A2
AZGP1: zinc-alpha-2-glycoprotein
CHCA: alpha-cyano-4-hydroxycinnamic acid
DTT: dithiothreitol
FFPE: formalin-fixed paraffin-embedded
LC: liquid chromatography
MS: mass spectrometry
PAGE: polyacrylamide gel electrophoresis
PCa: prostate cancer
PRDX1: peroxiredoxin-1
PSA: prostate-specific antigen
TFA: trifluoroacetic acid
TROG: Trans-Tasman Radiation Oncology Group
