# Supplementary material for: Proteins from formalin-fixed paraffin-embedded prostate cancer sections that predict the risk of metastatic disease
Source: Clin Proteomics. 2015 Sep 16;12(1):24. doi: 10.1186/s12014-015-9096-3 (PMC4574128; doi:10.1186/s12014-015-9096-3)
Supplement: Supplementary file 1 — Additional file 1: Two-dimensional electrophoresis of proteins extracted from FFPE tissue. [file 12014_2015_9096_MOESM1_ESM.pdf]

Additional file 1: Two-dimensional electrophoresis of proteins extracted from prostate FFPE tissue. Two-dimensional electrophoresis pI 3-10 (A) and 4-7 (B) separation of total protein extracted from FFPE prostate tumour tissue. Gels were fixed for 90 min at room temperature in 50% ethanol:3% orthophosphoric acid, washed 3x with ddH_2O, and stained overnight in 34% methanol (v/v), 17% ammonium sulfate (w/v), 3% ortho-phosphoric acid (v/v), and 0.05% Coomassie Brilliant Blue G-250. MS/MS identifications of numbered spots are summarized in Additional file 3.

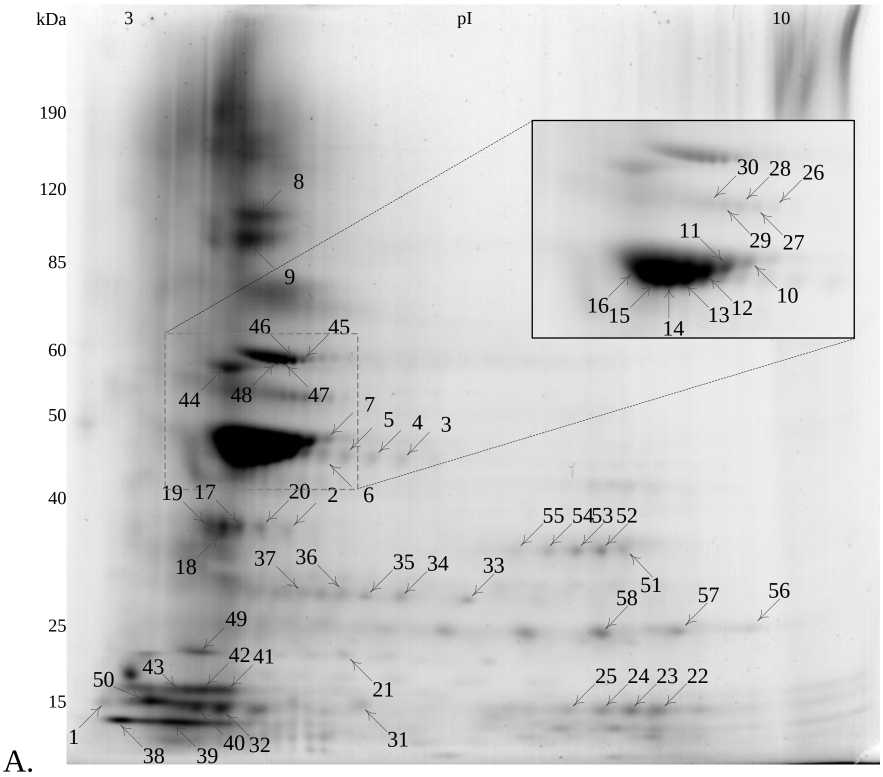

kDa
3
pI
10
190
120
85
60
50
40
25
15
8
9
30 28 26
29 27
11
10
16 15 14 13 12
46 45
44 48 47
7
5 4 3
2 6
19 17 20
18
55 54 53 52
51
37 36 35 34 33
58 57 56
49
43 42 41
50
21
25 24 23 22
1
38 39 40 32
31

A.

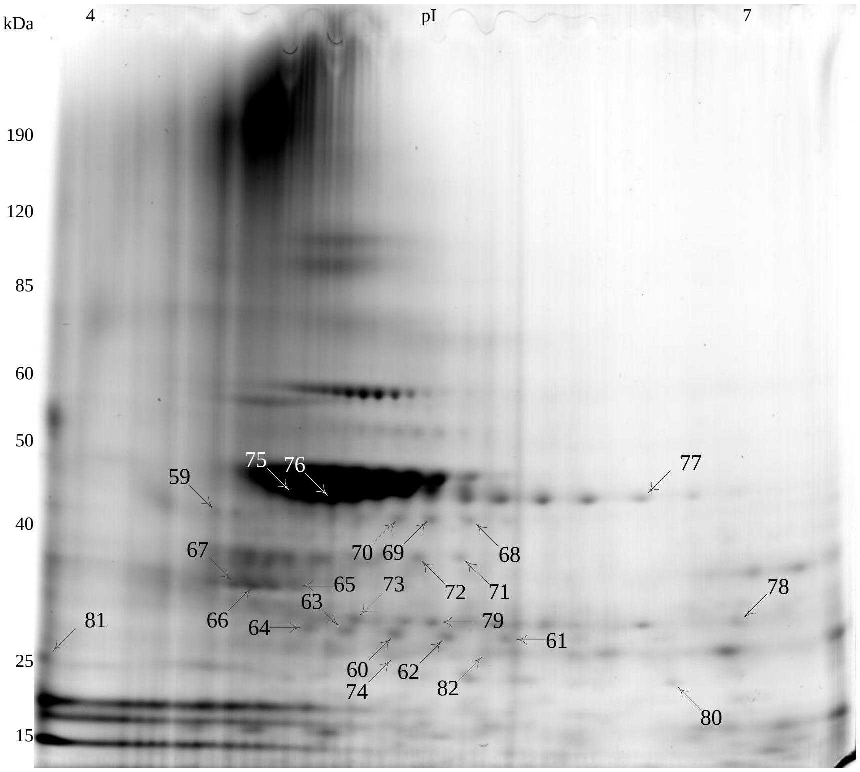

kDa
4
pI
7
190
120
85
60
50
40
25
15
75 76
59
77
70 69
68
67
65
73
72 71
78
81
66
64
63
79
61
60
62
74
82
80

B.
