# Supplementary material for: Proteins from formalin-fixed paraffin-embedded prostate cancer sections that predict the risk of metastatic disease
Source: Clin Proteomics. 2015 Sep 16;12(1):24. doi: 10.1186/s12014-015-9096-3 (PMC4574128; doi:10.1186/s12014-015-9096-3)

Additional file 2. Two-dimensional Western blot analysis of PSA. Proteins extracted from FFPE prostate tumour tissue were separated by two-dimensional electrophoresis, transferred to Hybond-LFP membrane and probed for PSA. Detection with AlexaFluor-labelled second antibodies was captured using a FLA-5100 scanner.

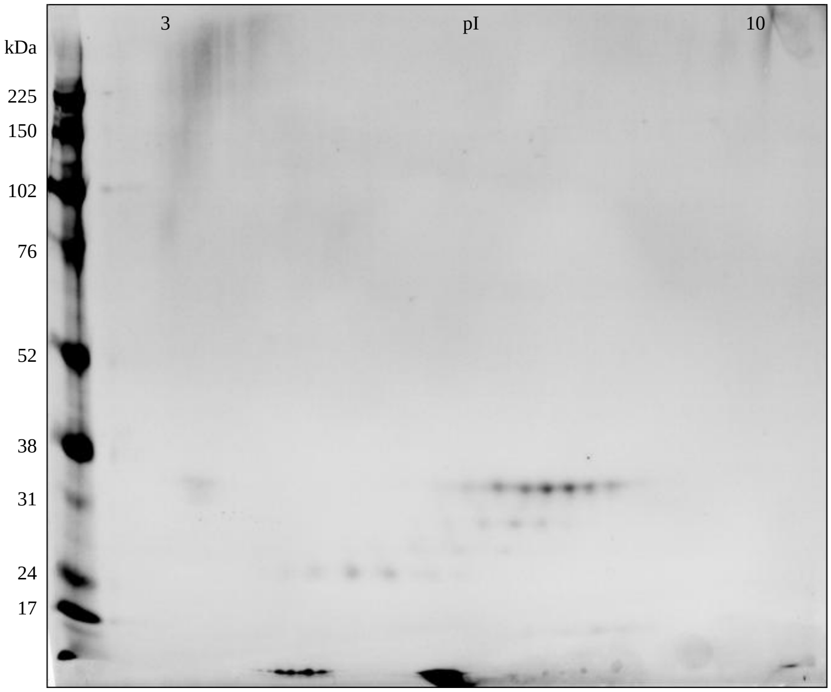

Supplement: Supplementary file 2 — Additional file 2: Two-dimensional Western blot analysis of PSA. [file 12014_2015_9096_MOESM2_ESM.pdf]
