# Supplementary material for: Proteins from formalin-fixed paraffin-embedded prostate cancer sections that predict the risk of metastatic disease
Source: Clin Proteomics. 2015 Sep 16;12(1):24. doi: 10.1186/s12014-015-9096-3 (PMC4574128; doi:10.1186/s12014-015-9096-3)
Supplement: Supplementary file 3 — Additional file 3: Summary of proteins identified by MALDI MS/MS of spots excised from 2DE gels. [file 12014_2015_9096_MOESM3_ESM.docx]

**Additional file 3. Summary of proteins identified by MALDI MS/MS of spots excised from 2DE gels**

| Spot^[[1]](#endnote-1)^ | Protein | Uniprot | Mass (Da) | pI | # Matched peptides | % Coverage | Mascot Score | Subcellular location |
| --- | --- | --- | --- | --- | --- | --- | --- | --- |
| 1 | 40S ribosomal protein S20 isoform 1 | B4DW28 | 16.2 | 3 | 8 | 45 | 73 | Cytoplasm |
| 2 | Actin, alpha cardiac muscle 1 proprotein2 | P68032 | 42.3 | 5.1 | 8 | 25 | 91 | Cytoplasm |
| 3 | Actin, alpha cardiac muscle 1 proprotein2 | P68032 | 42.3 | 5.1 | 17 | 41 | 174 | Cytoplasm |
| 4 | Actin, alpha cardiac muscle 1 proprotein2 | P68032 | 42.3 | 5.1 | 18 | 55 | 178 | Cytoplasm |
| 5 | Actin, alpha cardiac muscle 1 proprotein2 | P68032 | 42.3 | 5.1 | 15 | 41 | 146 | Cytoplasm |
| 6 | Actin, alpha cardiac muscle 1 proprotein2 | P68032 | 42.3 | 5.1 | 12 | 33 | 137 | Cytoplasm |
| 7 | Actin, alpha cardiac muscle 1 proprotein2 | P68032 | 42.3 | 5.1 | 17 | 49 | 167 | Cytoplasm |
| 8 | Actin, alpha cardiac muscle 1 proprotein2 | P68032 | 42.3 | 5.1 | 15 | 46 | 154 | Cytoplasm |
| 9 | Actin, alpha cardiac muscle 1 proprotein2 | P68032 | 42.3 | 5.1 | 14 | 47 | 148 | Cytoplasm |
| 10 | Actin, alpha cardiac muscle 1 proprotein2 | P68032 | 42.3 | 5.1 | 18 | 49 | 187 | Cytoplasm |
| 11 | Actin, alpha cardiac muscle 1 proprotein2 | P68032 | 42.3 | 5.1 | 15 | 43 | 158 | Cytoplasm |
| 12 | Actin, alpha cardiac muscle 1 proprotein2 | P68032 | 42.3 | 5.1 | 14 | 41 | 132 | Cytoplasm |
| 13 | Actin, alpha cardiac muscle 1 proprotein2 | P68032 | 42.3 | 5.1 | 14 | 41 | 128 | Cytoplasm |
| 14 | Actin, alpha cardiac muscle 1 proprotein2 | P68032 | 42.3 | 5.1 | 17 | 51 | 184 | Cytoplasm |
| 15 | Actin, alpha cardiac muscle 1 proprotein2 | P68032 | 42.3 | 5.1 | 13 | 41 | 137 | Cytoplasm |
| 16 | Actin, alpha cardiac muscle 1 proprotein2 | P68032 | 42.3 | 5.1 | 15 | 49 | 164 | Cytoplasm |
| 17 | Actin, alpha, cardiac muscle, isoform CRA_c2 | P68032 | 30.5 | 5.1 | 8 | 34 | 85 | Cytoplasm |
| 18 | Actin, alpha, cardiac muscle, isoform CRA_c2 | P68032 | 30.5 | 5.1 | 9 | 45 | 93 | Cytoplasm |
| 18 | Chain A, Human Annexin V With Incorporated Methionine Analogue Azidohomoalanine2,3 | P08758 | 35.8 | 4.3 | 10 | 36 | 98 | Cytoplasm |
| 19 | Actin, alpha, cardiac muscle, isoform CRA_c2 | P68032 | 30.5 | 5.1 | 9 | 45 | 94 | Cytoplasm |
| 19 | Chain A, Human Annexin V With Incorporated Methionine Analogue Azidohomoalanine2, 3 | P08758 | 35.8 | 4.3 | 8 | 26 | 71 | Cytoplasm |
| 20 | Actin, gamma-enteric smooth muscle isoform 2 precursor2 | P63267 | 37.5 | 5.36 | 7 | 28 | 69 | Cytoplasm |
| 21 | Adenine phosphoribosyltransferase isoform b2 | P07741 | 14.6 | 5.7 | 7 | 44 | 101 | Cytoplasm |
| 22 | Chain A, Refined Solution Structure Of Human Profilin I2 | P07737 | 15.0 | 8.1 | 6 | 43 | 77 | Cytoplasm |
| 22 | Chain B, Oxygen Affinity Modulation By The N-Termini Of The Beta Chains In Human And Bovine Hemoglobin2 | P68871 | 15.9 | 7.2 | 6 | 57 | 76 | Secreted |
| 23 | Chain B, Oxygen Affinity Modulation By The N-Termini Of The Beta Chains In Human And Bovine Hemoglobin2 | P68871 | 15.9 | 7.2 | 6 | 57 | 73 | Secreted |
| 24 | Chain B, Oxygen Affinity Modulation By The N-Termini Of The Beta Chains In Human And Bovine Hemoglobin2 | P68871 | 15.9 | 7.2 | 8 | 77 | 113 | Secreted |
| 25 | Chain B, Oxygen Affinity Modulation By The N-Termini Of The Beta Chains In Human And Bovine Hemoglobin2 | P68871 | 15.9 | 7.2 | 7 | 66 | 100 | Secreted |
| 26 | Cytokeratin 18 | P05783 | 47.3 | 5.5 | 16 | 37 | 155 | Cytoplasm |
| 27 | Cytokeratin 18 | P05783 | 47.3 | 5.5 | 18 | 40 | 191 | Cytoplasm |
| 28 | Cytokeratin 18 | P05783 | 47.3 | 5.5 | 19 | 40 | 196 | Cytoplasm |
| 29 | Cytokeratin 18 | P05783 | 47.3 | 5.5 | 19 | 40 | 184 | Cytoplasm |
| 30 | Cytokeratin 18 | P05783 | 47.3 | 5.5 | 16 | 38 | 149 | Cytoplasm |
| 31 | Fatty acid-binding protein, epidermal | Q01469 | 15.5 | 5.8 | 8 | 49 | 102 | Cytoplasm |
| 32 | hCG2039036 | EAW80467 | 13.6 | 4.4 | 6 | 63 | 73 | Unknown |
| 33 | Heat shock protein beta-12, 3 | P04792 | 22.4 | 6.5 | 8 | 37 | 97 | Cytoplasm |
| 34 | Heat shock protein beta-12, 3 | P04792 | 22.4 | 6.5 | 8 | 37 | 98 | Cytoplasm |
| 35 | Heat shock protein beta-12, 3 | P04792 | 22.8 | 6.5 | 8 | 39 | 102 | Cytoplasm |
| 36 | Heat shock protein beta-12,3 | P04792 | 22.4 | 6.5 | 6 | 30 | 70 | Cytoplasm |
| 37 | Heat shock protein beta-12, 3 | P04792 | 22.4 | 6.5 | 6 | 32 | 72 | Cytoplasm |
| 38 | HIST2H4B protein2, 3 | P62805 | 11.4 | 3.1 | 11 | 68 | 172 | Nucleus |
| 39 | HIST2H4B protein2, 3 | P62805 | 11.4 | 3.1 | 10 | 57 | 72 | Nucleus |
| 40 | Histone H2A type 1-H2, 3 | Q96KK5 | 13.9 | 4.2 | 6 | 40 | 73 | Nucleus |
| 41 | Histone H2B type 2-F isoform b | B4DLA9 | 14.8 | 4.5 | 7 | 48 | 80 | Nucleus |
| 42 | Histone H2B type 2-F isoform b | B4DLA9 | 14.8 | 4.5 | 8 | 52 | 84 | Nucleus |
| 42 | Unnamed protein product | B4DEB1 | 14.2 | 4.3 | 8 | 43 | 82 | Nucleus |
| 43 | Histone H2B type 2-F isoform b | B4DLA9 | 14.8 | 4.5 | 9 | 53 | 71 | Nucleus |
| 44 | Mitochondrial ATP synthase, H+ transporting F1 complex beta subunit | Q0QEN7 | 48.1 | 4.5 | 12 | 38 | 91 | Mitochondrion |
| 44 | Tubulin, beta2 | P07437 | 50.1 | 4.7 | 22 | 40 | 211 | Cytoplasm |
| 45 | Desmin3 | P17661 | 53.6 | 5.1 | 23 | 56 | 222 | Cytoplasm |
| 46 | Desmin3 | P17661 | 53.6 | 5.1 | 24 | 60 | 297 | Cytoplasm |
| 47 | Desmin3 | P17661 | 53.6 | 5.1 | 24 | 60 | 288 | Cytoplasm |
| 48 | Desmin3 | P17661 | 53.6 | 5.1 | 21 | 53 | 238 | Cytoplasm |
| 49 | Myosin regulatory light polypeptide 9 isoform a2, 3 | P24844 | 19.9 | 4.1 | 8 | 52 | 104 | Cytoplasm |
| 50 | Myosin, light polypeptide 6, alkali, smooth muscle and non-muscle, isoform CRA_c2, 3 | P60660 | 18.3 | 3.5 | 12 | 77 | 148 | Cytoplasm |
| 51 | Prostate specific antigen2 | P07288 | 25.6 | 7.9 | 8 | 25 | 53 | Secreted |
| 52 | Prostate-specific antigen2 | P07288 | 25.6 | 7.9 | 11 | 30 | 59 | Secreted |
| 53 | Prostate specific antigen2 | P07288 | 25.6 | 7.9 | 7 | 30 | 71 | Secreted |
| 54 | Prostate specific antigen2 | P07288 | 25.6 | 7.9 | 8 | 30 | 68 | Secreted |
| 55 | Prostate specific antigen2 | P07288 | 25.6 | 7.9 | 5 | 28 | 50 | Secreted |
| 56 | Transgelin2 | Q01995 | 22.5 | 9 | 13 | 67 | 149 | Cytoplasm |
| 57 | Transgelin2 | Q01995 | 22.5 | 9 | 9 | 58 | 105 | Cytoplasm |
| 58 | Transgelin2 | Q01995 | 22.5 | 9 | 11 | 58 | 128 | Cytoplasm |
| 59 | 40S ribosomal protein SA | P08865 | 32.9 | 4.79 | 10 | 42 | 103 | Nucleus |
| 60 | ACTB protein, partial^[[2]](#endnote-2)^, ^[[3]](#endnote-3)^ | P60709 | 40.5 | 5.55 | 9 | 24 | 66 | Cytoplasm |
| 61 | Actin, alpha, cardiac muscle, isoform CRA_b2 | P68032 | 22.8 | 5.1 | 9 | 51 | 82 | Cytoplasm |
| 62 | Actin, alpha, cardiac muscle, isoform CRA_b2 | P68032 | 22.8 | 5.1 | 9 | 44 | 99 | Cytoplasm |
| 63 | Actin, alpha cardiac muscle 1 proprotein2 | P68032 | 42.3 | 5.1 | 11 | 30 | 100 | Cytoplasm |
| 64 | Actin, alpha cardiac muscle 1 proprotein2 | P68032 | 42.3 | 5.1 | 11 | 33 | 95 | Cytoplasm |
| 65 | Actin, alpha, cardiac muscle, isoform CRA_c2 | P68032 | 30.5 | 5.1 | 9 | 36 | 90 | Cytoplasm |
| 66 | Actin, alpha, cardiac muscle, isoform CRA_c2 | P68032 | 30.5 | 5.1 | 7 | 30 | 76 | Cytoplasm |
| 67 | Actin, alpha, cardiac muscle, isoform CRA_c2 | P68032 | 30.5 | 5.1 | 8 | 34 | 77 | Cytoplasm |
| 68 | Actin, alpha cardiac muscle 1 proprotein2 | P68032 | 42.3 | 5.1 | 9 | 30 | 80 | Cytoplasm |
| 69 | Actin, alpha cardiac muscle 1 proprotein2 | P68032 | 42.3 | 5.1 | 11 | 31 | 95 | Cytoplasm |
| 70 | Actin, alpha cardiac muscle 1 proprotein2 | P68032 | 42.3 | 5.1 | 9 | 30 | 83 | Cytoplasm |
| 71 | Actin, alpha cardiac muscle 1 proprotein2 | P68032 | 42.3 | 5.1 | 11 | 31 | 100 | Cytoplasm |
| 72 | Actin, alpha cardiac muscle 1 proprotein2 | P68032 | 42.3 | 5.1 | 10 | 31 | 85 | Cytoplasm |
| 73 | Actin, alpha, cardiac muscle, isoform CRA_c2 | P68032 | 30.5 | 5.1 | 7 | 30 | 71 | Cytoplasm |
| 74 | Actin, alpha, cardiac muscle, isoform CRA_b2 | P68032 | 22.8 | 5.1 | 8 | 45 | 68 | Cytoplasm |
| 75 | Actin, alpha cardiac muscle 1 proprotein2 | P68032 | 42.3 | 5.1 | 12 | 41 | 120 | Cytoplasm |
| 76 | Actin, alpha cardiac muscle 1 proprotein2 | P68032 | 42.3 | 5.1 | 11 | 33 | 108 | Cytoplasm |
| 77 | Actin, alpha cardiac muscle 1 proprotein2 | P68032 | 42.3 | 5.1 | 11 | 37 | 109 | Cytoplasm |
| 78 | Heat shock protein beta-12, 3 | P04792 | 22.4 | 6.5 | 7 | 33 | 80 | Cytoplasm |
| 79 | Heat shock protein beta-12, 3 | P04792 | 22.4 | 6.5 | 8 | 37 | 91 | Cytoplasm |
| 80 | Heat shock beta-62 | O14558 | 16.9 | 6.6 | 7 | 36 | 91 | Cytoplasm |
| 81 | Myosin regulatory light polypeptide 9 isoform a2, 3 | P24844 | 19.9 | 4.1 | 8 | 52 | 91 | Cytoplasm |
| 82 | Peroxiredoxin-22 | P32119 | 22.0 | 5.66 | 8 | 34 | 88 | Cytoplasm |

1. Protein spot numbers are from Additional file 1. [↑](#endnote-ref-1)
2. Also identified by LC-MS/MS. [↑](#endnote-ref-2)
3. Also identified by Gel-MS/MS. [↑](#endnote-ref-3)
