# Supplementary material for: Proteins from formalin-fixed paraffin-embedded prostate cancer sections that predict the risk of metastatic disease
Source: Clin Proteomics. 2015 Sep 16;12(1):24. doi: 10.1186/s12014-015-9096-3 (PMC4574128; doi:10.1186/s12014-015-9096-3)
Supplement: Supplementary file 4 — Additional file 4: Summary of proteins identified in FFPE prostate tumor extracts using Gel-MS/MS. [file 12014_2015_9096_MOESM4_ESM.docx]

**Additional file 4. Summary of proteins identified in FFPE prostate tumour extracts using Gel-MS/MS.**

| Protein | Uniprot | Mass (Da) | # Matched peptides | % Coverage | Paragon Score | Subcellular location |
| --- | --- | --- | --- | --- | --- | --- |
| Actin, alpha skeletal muscle^[[1]](#endnote-1)^ | P68133 | 42.1 | 31 | 63.4 | 35.68 | Cytoplasm/cytoskeleton |
| Actin, cytoplasmic 11,^[[2]](#endnote-2)^ | P60709 | 41.7 | 27 | 55.7 | 35.4 | Cytoplasm/cytoskeleton |
| Actin, cytoplasmic 21 | P63261 | 41.8 | 27 | 55.7 | 35.4 | Cytoplasm/cytoskeleton |
| Annexin A51,2 | P08758 | 35.9 | 6 | 21.9 | 10 | Cytoplasm |
| Calmodulin1 | P62158 | 16.8 | 1 | 18.1 | 1.71 | Cytoplasm |
| Creatine kinase B-type | P12277 | 42.6 | 1 | 15.7 | 2 | Cytoplasm |
| Cyclic AMP-responsive element-binding protein 3-like protein 2 | Q70SY1 | 57.4 | 1 | 9.4 | 1.52 | Nucleus |
| Desmin2 | P17661 | 53.5 | 3 | 33.0 | 7.14 | Mitochondrion |
| Heat shock protein beta-11,2 | P04792 | 22.8 | 3 | 24.9 | 6.44 | Nucleus |
| Heterogeneous nuclear ribonucleoproteins A2/B1 | P22626 | 37.4 | 1 | 15.0 | 1.4 | Nucleus |
| Histone H2A type 11 | P0C0S8 | 14.1 | 6 | 53.1 | 10.08 | Nucleus |
| Histone H2A type 1-A1 | Q96QV6 | 14.2 | 6 | 57.2 | 10.01 | Nucleus |
| Histone H2A type 1-B/E1 | P04908 | 14.1 | 5 | 53.1 | 8.28 | Nucleus |
| Histone H2A type 1-C1 | Q93077 | 14.1 | 6 | 40.0 | 10.08 | Nucleus |
| Histone H2A type 1-D1 | P20671 | 14.1 | 5 | 53.1 | 8.28 | Nucleus |
| Histone H2A type 1-H1,2 | Q96KK5 | 13.9 | 6 | 53.9 | 10.08 | Nucleus |
| Histone H2A type 1-J1 | Q99878 | 13.9 | 6 | 53.9 | 10.08 | Nucleus |
| Histone H2A type 2-A1 | Q6FI13 | 14.1 | 6 | 40.0 | 10.08 | Nucleus |
| Histone H2A type 2-B1 | Q8IUE6 | 14.0 | 3 | 32.3 | 3.72 | Nucleus |
| Histone H2A type 2-C1 | Q16777 | 14.0 | 6 | 40.3 | 10.08 | Nucleus |
| Histone H2A type 31 | Q7L7L0 | 14.1 | 5 | 40.0 | 8.28 | Nucleus |
| Histone H2A.J1 | Q9BTM1 | 14.0 | 6 | 40.3 | 10.08 | Nucleus |
| Histone H2A.V1 | Q71UI9 | 13.5 | 2 | 28.9 | 4 | Nucleus |
| Histone H2A.Z1 | P0C0S5 | 13.6 | 2 | 12.5 | 4 | Nucleus |
| Histone H2AX1 | P16104 | 15.1 | 5 | 36.4 | 8.51 | Nucleus |
| Histone H2B type F-S1 | P57053 | 13.9 | 7 | 47.6 | 6.16 | Nucleus |
| Histone H3.11 | P68431 | 15.4 | 5 | 47.8 | 5.63 | Nucleus |
| Histone H3.21 | Q71DI3 | 15.4 | 5 | 47.8 | 5.63 | Nucleus |
| Histone H3.31 | P84243 | 15.3 | 6 | 48.5 | 7.63 | Nucleus |
| Histone H41,2 | P62805 | 11.4 | 11 | 70.9 | 18.63 | Nucleus |
| Myosin light polypeptide 61,2 | P60660 | 16.9 | 10 | 60.3 | 15 | Cytoplasm |
| Myosin regulatory light chain 12A1 | P19105 | 19.8 | 2 | 19.3 | 4 | Mitochondrion |
| Myosin regulatory light chain 12B1 | O14950 | 19.8 | 2 | 11.6 | 4 | Mitochondrion |
| Myosin regulatory light polypeptide 91,2 | P24844 | 19.8 | 5 | 29.6 | 8 | Mitochondrion |
| Polyubiquitin-B1 | P0CG47 | 25.8 | 1 | 17.0 | 2 | Cytoplasm |
| Polyubiquitin-C1 | P0CG48 | 77.0 | 1 | 17.1 | 2 | Cytoplasm |
| POTE ankyrin domain family member F | A5A3E0 | 121.4 | 9 | 9.8 | 9.4 | Cytoplasm |
| Serum albumin1 | P02768 | 69.4 | 37 | 54.8 | 62.75 | Extracellular |
| Tropomyosin alpha-1 chain1 | P09493 | 32.7 | 1 | 3.5 | 2 | Cytoplasm |
| Tropomyosin alpha-3 chain1 | P06753 | 33.0 | 1 | 3.5 | 2 | Cytoplasm |
| Tropomyosin alpha-4 chain1 | P67936 | 28.5 | 1 | 4.0 | 2 | Cytoplasm |
| Tropomyosin beta chain1 | P07951 | 32.9 | 1 | 3.5 | 2 | Cytoplasm |
| Tubulin alpha-1A chain1 | Q71U36 | 50.1 | 1 | 12.6 | 2 | Cytoplasm/cytoskeleton |
| Tubulin alpha-1B chain1 | P68363 | 50.2 | 1 | 12.6 | 2 | Cytoplasm/cytoskeleton |
| Tubulin alpha-1C chain1 | Q9BQE3 | 49.9 | 1 | 8.9 | 2 | Cytoplasm/cytoskeleton |
| Ubiquitin-40S ribosomal protein S27a1 | P62979 | 18.0 | 1 | 8.3 | 2 | Cytoplasm/nucleus |
| Ubiquitin-60S ribosomal protein L401 | P62987 | 14.7 | 1 | 10.2 | 2 | Extracellular |

1. Also identified by LC-MS/MS. [↑](#endnote-ref-1)
2. Also identified by 2DE-MALDI. [↑](#endnote-ref-2)
