# Supplementary material for: Proteins from formalin-fixed paraffin-embedded prostate cancer sections that predict the risk of metastatic disease
Source: Clin Proteomics. 2015 Sep 16;12(1):24. doi: 10.1186/s12014-015-9096-3 (PMC4574128; doi:10.1186/s12014-015-9096-3)
Supplement: Supplementary file 5 — Additional file 5: Summary of proteins identified in FFPE prostate tumor extracts using LC-MS/MS. [file 12014_2015_9096_MOESM5_ESM.docx]

**Additional file 5. Summary of proteins identified in FFPE prostate tumour extracts using LC-MS/MS.**

| Protein | Uniprot | Mass (Da) | # Matched peptides | % Coverage | Paragon Score | Subcellular location |
| --- | --- | --- | --- | --- | --- | --- |
| 10 kDa heat shock protein, mitochondrial | P61604 | 10.9 | 1 | 19.6 | 3.16 | Mitochondrion |
| 14-3-3 Protein beta/alpha | P31946 | 28.1 | 1 | 9.8 | 1.52 | Cytoplasm |
| 14-3-3 Protein epsilon | P62258 | 29.2 | 1 | 12.9 | 1.65 | Cytoplasm |
| 14-3-3 Protein eta | Q04917 | 28.2 | 1 | 4.1 | 1.52 | Cytoplasm |
| 14-3-3 Protein gamma | P61981 | 28.3 | 1 | 9.7 | 1.88 | Cytoplasm |
| 14-3-3 Protein sigma | P31947 | 27.8 | 1 | 4.0 | 1.52 | Cytoplasm |
| 14-3-3 Protein theta | P27348 | 27.8 | 1 | 9.8 | 1.52 | Cytoplasm |
| 14-3-3 Protein zeta/delta | P63104 | 27.7 | 2 | 9.8 | 3.52 | Cytoplasm |
| 40S Ribosomal protein S18 | P62269 | 17.7 | 1 | 19.7 | 1.53 | Cytoplasm |
| 40S Ribosomal protein S19 | P39019 | 16.1 | 1 | 6.2 | 2 | Cytoplasm |
| 40S Ribosomal protein S24 | P62847 | 15.4 | 1 | 13.5 | 2 | Cytoplasm |
| 60S Ribosomal protein L12 | P30050 | 17.8 | 1 | 9.1 | 2 | Cytoplasm |
| 60S Ribosomal protein L18 | Q07020 | 21.6 | 1 | 14.9 | 1.4 | Cytoplasm |
| 60S Ribosomal protein L27a | P46776 | 16.6 | 1 | 14.2 | 2 | Cytoplasm |
| 60S Ribosomal protein L35a | P18077 | 12.5 | 1 | 24.5 | 2.01 | Cytoplasm |
| Acid ceramidase | Q13510 | 44.7 | 1 | 7.3 | 2.95 | Extracellular |
| Actin, alpha cardiac muscle 1^[[1]](#endnote-1)^ | P68032 | 42.0 | 40 | 63.4 | 41.49 | Cytoplasm/cytoskeleton |
| Actin, alpha skeletal muscle^[[2]](#endnote-2)^ | P68133 | 42.1 | 31 | 63.4 | 35.68 | Cytoplasm/cytoskeleton |
| Actin, aortic smooth muscle | P62736 | 42.0 | 43 | 63.4 | 43.19 | Cytoplasm/cytoskeleton |
| Actin, cytoplasmic 14,5 | P60709 | 41.7 | 27 | 55.7 | 35.4 | Cytoplasm/cytoskeleton |
| Actin, cytoplasmic 25 | P63261 | 41.8 | 27 | 55.7 | 35.4 | Cytoplasm/cytoskeleton |
| Actin, gamma-enteric smooth muscle4 | P63267 | 41.9 | 42 | 68.1 | 43.2 | Cytoplasm/cytoskeleton |
| Acyl-CoA-binding protein | P07108 | 10.0 | 1 | 32.2 | 1.74 | Cytoplasm |
| Adenine phosphoribosyltransferase4 | P07741 | 19.6 | 1 | 13.3 | 1.89 | Cytoplasm |
| Alpha-1-acid glycoprotein 1 | P02763 | 23.5 | 2 | 22.9 | 3.4 | Extracellular |
| Alpha-1-acid glycoprotein 2 | P19652 | 23.6 | 1 | 15.4 | 2 | Extracellular |
| Alpha-1-antitrypsin | P01009 | 46.7 | 3 | 6.9 | 6 | Extracellular |
| Annexin A2 | P07355 | 38.6 | 1 | 11.8 | 2.32 | Cytoplasm |
| Annexin A54,5 | P08758 | 35.9 | 6 | 21.9 | 10 | Cytoplasm |
| Annexin A6 | P08133 | 75.9 | 1 | 3.9 | 2 | Cytoplasm |
| Anterior gradient protein 2 homolog | O95994 | 20.0 | 1 | 6.3 | 2 | Extracellular |
| Apolipoprotein D | P05090 | 21.3 | 1 | 7.9 | 1.7 | Extracellular |
| ATP synthase subunit beta, mitochondrial | P06576 | 56.6 | 1 | 5.5 | 3.15 | Mitochondrion |
| Biglycan | P21810 | 41.7 | 4 | 15.8 | 8 | Mitochondrion |
| Calmodulin5 | P62158 | 16.8 | 1 | 18.1 | 1.71 | Cytoplasm |
| Cathepsin D | P07339 | 44.6 | 3 | 14.3 | 5.41 | Extracellular |
| Cathepsin G | P08311 | 28.8 | 2 | 24.7 | 4.01 | Extracellular |
| CD59 glycoprotein | P13987 | 14.2 | 1 | 9.4 | 2 | Extracellular |
| Chymase | P23946 | 27.3 | 1 | 5.7 | 2 | Extracellular |
| Clusterin | P10909 | 52.5 | 1 | 2.7 | 2 | Extracellular |
| Collagen alpha-1(VI) chain | P12109 | 108.5 | 1 | 7.2 | 2.23 | Mitochondrion |
| Collagen alpha-2(I) chain | P08123 | 129.3 | 1 | 6.0 | 3 | Extracellular |
| Collagen alpha-2(VI) chain | P12110 | 108.6 | 1 | 9.7 | 2.59 | Extracellular |
| Collagen alpha-3(VI) chain | P12111 | 343.7 | 1 | 4.3 | 2.38 | Extracellular |
| Complement C4-A | P0C0L4 | 192.8 | 10 | 11.6 | 21.49 | Extracellular |
| Complement C4-B | P0C0L5 | 192.8 | 10 | 11.6 | 21.49 | Extracellular |
| Cystatin-B | P04080 | 11.1 | 1 | 12.2 | 2 | Cytoplasm |
| Cysteine and glycine-rich protein 1 | P21291 | 20.6 | 1 | 15.5 | 2 | Nucleus |
| D-dopachrome decarboxylase | P30046 | 12.7 | 1 | 9.3 | 1.7 | Cytoplasm |
| Decorin | P07585 | 39.7 | 1 | 12.0 | 2.3 | Extracellular |
| Dermatopontin | Q07507 | 24.0 | 1 | 13.9 | 1.53 | Extracellular |
| Destrin | P60981 | 18.5 | 1 | 15.2 | 2.62 | Mitochondrion |
| Dipeptidyl peptidase 2 | Q9UHL4 | 54.3 | 2 | 13.4 | 4 | Plasma membrane |
| Extracellular superoxide dismutase [Cu-Zn] | P08294 | 25.9 | 1 | 4.6 | 2 | Extracellular |
| Ezrin | P15311 | 69.4 | 1 | 7.9 | 2.12 | Plasma membrane |
| Ferritin heavy chain | P02794 | 21.2 | 1 | 14.7 | 2.01 | Cytoplasm |
| Ferritin light chain | P02792 | 20.0 | 1 | 17.1 | 2.01 | Cytoplasm |
| Fibrillin-1 | P35555 | 312.2 | 1 | 1.3 | 1.7 | Nucleus |
| Fructose-bisphosphate aldolase A | P04075 | 39.4 | 2 | 11.5 | 3.18 | Cytoplasm |
| Fructose-bisphosphate aldolase C | P09972 | 39.5 | 1 | 1.9 | 1.4 | Cytoplasm |
| Galectin-1 | P09382 | 14.7 | 3 | 29.6 | 6 | Extracellular |
| Galectin-3-binding protein | Q08380 | 65.3 | 1 | 6.2 | 1.77 | Extracellular |
| Glyceraldehyde-3-phosphate dehydrogenase | P04406 | 36.1 | 3 | 20.3 | 5.79 | Cytoplasm |
| Granulins | P28799 | 63.5 | 1 | 3.9 | 2.85 | Extracellular |
| Growth/differentiation factor 15 | Q99988 | 34.1 | 1 | 14.0 | 2.64 | Extracellular |
| Haptoglobin | P00738 | 45.2 | 3 | 20.9 | 6.45 | Extracellular |
| Heat shock 70 kDa protein 1A/1B | P08107 | 70.1 | 2 | 10.3 | 4 | Cytoplasm |
| Heat shock 70 kDa protein 1-like | P34931 | 70.4 | 1 | 8.7 | 2.02 | Cytoplasm |
| Heat shock 70 kDa protein 6 | P17066 | 71.0 | 1 | 8.4 | 2.75 | Cytoplasm |
| Heat shock cognate 71 kDa protein | P11142 | 70.9 | 2 | 8.5 | 4.02 | Cytoplasm |
| Heat shock protein beta-14,5 | P04792 | 22.8 | 3 | 24.9 | 6.44 | Nucleus |
| Heat shock protein beta-64 | O14558 | 17.1 | 1 | 23.1 | 3.45 | Cytoplasm/nucleus |
| Heat shock-related 70 kDa protein 2 | P54652 | 70.0 | 2 | 6.4 | 4.02 | Cytoplasm |
| Heme-binding protein 2 | Q9Y5Z4 | 22.9 | 1 | 12.2 | 2 | Cytoplasm/Mitochondrion |
| Hemoglobin subunit alpha | P69905 | 15.3 | 3 | 28.2 | 4.01 | Cytoplasm |
| Hemoglobin subunit beta4 | P68871 | 16.0 | 9 | 65.3 | 10.02 | Cytoplasm |
| Hemopexin | P02790 | 51.7 | 2 | 13.6 | 5.14 | Extracellular |
| Hepatoma-derived growth factor | P51858 | 26.8 | 1 | 3.3 | 1.52 | Cytoplasm/nucleus |
| Hepatoma-derived growth factor-related protein 2 | Q7Z4V5 | 74.3 | 1 | 2.8 | 1.52 | Nucleus |
| Hepatoma-derived growth factor-related protein 3 | Q9Y3E1 | 22.6 | 1 | 13.3 | 1.52 | Nucleus |
| Histone H2A type 15 | P0C0S8 | 14.1 | 6 | 53.1 | 10.08 | Nucleus |
| Histone H2A type 1-A5 | Q96QV6 | 14.2 | 6 | 57.2 | 10.01 | Nucleus |
| Histone H2A type 1-B/E5 | P04908 | 14.1 | 5 | 53.1 | 8.28 | Nucleus |
| Histone H2A type 1-C5 | Q93077 | 14.1 | 6 | 40.0 | 10.08 | Nucleus |
| Histone H2A type 1-D5 | P20671 | 14.1 | 5 | 53.1 | 8.28 | Nucleus |
| Histone H2A type 1-H4,5 | Q96KK5 | 13.9 | 6 | 53.9 | 10.08 | Nucleus |
| Histone H2A type 1-J5 | Q99878 | 13.9 | 6 | 53.9 | 10.08 | Nucleus |
| Histone H2A type 2-A5 | Q6FI13 | 14.1 | 6 | 40.0 | 10.08 | Nucleus |
| Histone H2A type 2-B5 | Q8IUE6 | 14.0 | 3 | 32.3 | 3.72 | Nucleus |
| Histone H2A type 2-C5 | Q16777 | 14.0 | 6 | 40.3 | 10.08 | Nucleus |
| Histone H2A type 35 | Q7L7L0 | 14.1 | 5 | 40.0 | 8.28 | Nucleus |
| Histone H2A.J5 | Q9BTM1 | 14.0 | 6 | 40.3 | 10.08 | Nucleus |
| Histone H2A.V5 | Q71UI9 | 13.5 | 2 | 28.9 | 4 | Nucleus |
| Histone H2A.Z5 | P0C0S5 | 13.6 | 2 | 12.5 | 4 | Nucleus |
| Histone H2AX5 | P16104 | 15.1 | 5 | 36.4 | 8.51 | Nucleus |
| Histone H2B type 1-B | P33778 | 14.0 | 8 | 40.5 | 8.16 | Nucleus |
| Histone H2B type 1-C/E/F/G/I | P62807 | 13.9 | 8 | 51.6 | 8.16 | Nucleus |
| Histone H2B type 1-D | P58876 | 13.9 | 8 | 51.6 | 8.16 | Nucleus |
| Histone H2B type 1-H | Q93079 | 13.9 | 8 | 51.6 | 8.16 | Nucleus |
| Histone H2B type 1-J | P06899 | 13.9 | 8 | 51.6 | 8.16 | Nucleus |
| Histone H2B type 1-K | O60814 | 13.9 | 8 | 51.6 | 8.16 | Nucleus |
| Histone H2B type 1-L | Q99880 | 14.0 | 8 | 51.6 | 8.16 | Nucleus |
| Histone H2B type 1-M | Q99879 | 14.0 | 8 | 40.5 | 8.16 | Nucleus |
| Histone H2B type 1-N | Q99877 | 13.9 | 8 | 51.6 | 8.16 | Nucleus |
| Histone H2B type 1-O | P23527 | 13.9 | 8 | 51.6 | 8.16 | Nucleus |
| Histone H2B type 2-E | Q16778 | 13.9 | 8 | 51.6 | 8.16 | Nucleus |
| Histone H2B type 2-F | Q5QNW6 | 13.9 | 8 | 40.5 | 8.16 | Nucleus |
| Histone H2B type 3-B | Q8N257 | 13.9 | 8 | 46.0 | 8 | Nucleus |
| Histone H2B type F-S5 | P57053 | 13.9 | 7 | 47.6 | 6.16 | Nucleus |
| Histone H3.15 | P68431 | 15.4 | 5 | 47.8 | 5.63 | Nucleus |
| Histone H3.1t | Q16695 | 15.5 | 5 | 47.8 | 5.63 | Nucleus |
| Histone H3.25 | Q71DI3 | 15.4 | 5 | 47.8 | 5.63 | Nucleus |
| Histone H3.35 | P84243 | 15.3 | 6 | 48.5 | 7.63 | Nucleus |
| Histone H44,5 | P62805 | 11.4 | 11 | 70.9 | 18.63 | Nucleus |
| HLA class II histocompatibility antigen, DRB1-4 beta chain | P13760 | 30.1 | 1 | 13.2 | 2.08 | Extracellular |
| Ig alpha-1 chain C region | P01876 | 37.7 | 3 | 9.6 | 5.7 | Extracellular |
| Ig alpha-2 chain C region | P01877 | 36.5 | 3 | 10.0 | 5.7 | Nucleus |
| Ig gamma-2 chain C region | P01859 | 35.9 | 1 | 13.8 | 2.52 | Mitochondrion |
| Ig gamma-3 chain C region | P01860 | 41.3 | 1 | 7.4 | 2.52 | Extracellular |
| Ig gamma-4 chain C region | P01861 | 35.9 | 1 | 8.6 | 2.01 | Mitochondrion |
| Ig heavy chain V-III region BRO | P01766 | 13.2 | 1 | 9.2 | 2 | Extracellular |
| Ig heavy chain V-III region BUT | P01767 | 12.4 | 1 | 16.5 | 2 | Extracellular |
| Ig heavy chain V-III region WEA | P01763 | 12.3 | 1 | 9.6 | 2 | Extracellular |
| Ig kappa chain C region | P01834 | 11.6 | 4 | 48.1 | 7.82 | Extracellular |
| Ig lambda-1 chain C regions | P0CG04 | 11.3 | 1 | 14.1 | 2 | Plasma membrane |
| Ig lambda-2 chain C regions | P0CG05 | 11.3 | 1 | 14.1 | 2 | Plasma membrane |
| Ig lambda-3 chain C regions | P0CG06 | 11.2 | 1 | 14.1 | 2 | Plasma membrane |
| Ig lambda-6 chain C region | P0CF74 | 11.3 | 1 | 14.1 | 2 | Plasma membrane |
| Immunoglobulin lambda-like polypeptide 5 | B9A064 | 23.1 | 1 | 7.0 | 2 | Extracellular |
| Isocitrate dehydrogenase [NADP] cytoplasmic | O75874 | 46.7 | 1 | 14.7 | 3.31 | Cytoplasm |
| Lactotransferrin | P02788 | 78.2 | 2 | 14.8 | 5.45 | Extracellular |
| Lumican | P51884 | 38.4 | 3 | 11.2 | 6.17 | Extracellular |
| Lysosomal alpha-glucosidase | P10253 | 105.3 | 3 | 10.0 | 6.76 | Lysosome |
| Lysosome-associated membrane glycoprotein 2 | P13473 | 45.0 | 2 | 4.9 | 3.3 | Extracellular |
| Lysozyme C | P61626 | 16.5 | 1 | 8.1 | 2 | Extracellular |
| Macrophage migration inhibitory factor | P14174 | 12.5 | 2 | 17.4 | 3.3 | Cytoplasm/nucleus |
| Malate dehydrogenase, cytoplasmic | P40925 | 36.4 | 1 | 7.5 | 2.03 | Cytoplasm |
| MAM and LDL-receptor class A domain-containing protein | Q5VYJ5 | 164.0 | 1 | 3.0 | 1.52 | Plasma membrane |
| Membrane-associated progesterone receptor component 1 | O00264 | 21.7 | 1 | 7.2 | 2 | Nucleus |
| Mimecan | P20774 | 33.9 | 1 | 17.8 | 2.01 | Extracellular |
| Moesin | P26038 | 67.8 | 1 | 5.7 | 2.12 | Plasma membrane |
| Mucin-12 | Q9UKN1 | 558.2 | 1 | 2.0 | 1.31 | Plasma membrane |
| Myomegalin | Q5VU43 | 265.1 | 1 | 3.5 | 1.72 | Cytoplasm/cytoskeleton |
| Myosin light chain 1/3, skeletal muscle isoform | P05976 | 21.1 | 2 | 14.9 | 2 | Cytoplasm |
| Myosin light chain 3 | P08590 | 21.9 | 2 | 8.2 | 2 | Cytoplasm |
| Myosin light chain kinase, smooth muscle | Q15746 | 210.7 | 1 | 2.8 | 1.7 | Cytoplasm |
| Myosin light polypeptide 64,5 | P60660 | 16.9 | 10 | 60.3 | 15 | Cytoplasm |
| Myosin regulatory light chain 12A5 | P19105 | 19.8 | 2 | 19.3 | 4 | Mitochondrion |
| Myosin regulatory light chain 12B5 | O14950 | 19.8 | 2 | 11.6 | 4 | Mitochondrion |
| Myosin regulatory light polypeptide 94,5 | P24844 | 19.8 | 5 | 29.6 | 8 | Mitochondrion |
| Neutrophil defensin 1 | P59665 | 10.2 | 2 | 19.1 | 4 | Extracellular |
| Neutrophil defensin 3 | P59666 | 10.2 | 2 | 19.1 | 4 | Extracellular |
| Pancreatic secretory granule membrane major glycoprotein GP2 | P55259 | 59.5 | 2 | 8.8 | 4.27 | Plasma membrane |
| PC4 and SFRS1-interacting protein | O75475 | 60.1 | 1 | 6.2 | 1.52 | Nucleus |
| Peptidyl-prolyl cis-trans isomerase A | P62937 | 18.0 | 2 | 19.4 | 4 | Cytoplasm |
| Peptidyl-prolyl cis-trans isomerase A-like 4A/B/C | Q9Y536 | 18.2 | 1 | 8.5 | 2 | Cytoplasm |
| Peptidyl-prolyl cis-trans isomerase A-like 4D | F5H284 | 18.2 | 1 | 14.0 | 2.01 | Cytoplasm |
| Peptidyl-prolyl cis-trans isomerase FKBP1A | P62942 | 12.0 | 1 | 19.4 | 2 | Cytoplasm |
| Peroxiredoxin-1 | Q06830 | 22.1 | 1 | 5.5 | 1.52 | Cytoplasm |
| Peroxiredoxin-24 | P32119 | 21.9 | 2 | 14.7 | 3.52 | Cytoplasm |
| Peroxiredoxin-6 | P30041 | 25.0 | 3 | 21.4 | 6 | Mitochondrion |
| Phosphatidylethanolamine-binding protein 1 | P30086 | 21.1 | 1 | 14.4 | 2.01 | Cytoplasm |
| Phospholipase A2, membrane associated | P14555 | 16.1 | 5 | 52.1 | 10.62 | Extracellular |
| Polyubiquitin-B5 | P0CG47 | 25.8 | 1 | 17.0 | 2 | Cytoplasm |
| Polyubiquitin-C5 | P0CG48 | 77.0 | 1 | 17.1 | 2 | Cytoplasm |
| Profilin-14 | P07737 | 15.1 | 2 | 30.0 | 3.72 | Cytoplasm |
| Prolargin | P51888 | 43.8 | 3 | 19.6 | 6.9 | Extracellular |
| Prostate-specific antigen4 | P07288 | 28.7 | 10 | 42.1 | 13.52 | Extracellular |
| Prostatic acid phosphatase | P15309 | 44.6 | 10 | 28.5 | 17.56 | Extracellular |
| Proteasome activator complex subunit 2 | Q9UL46 | 27.4 | 1 | 5.4 | 2 | Cytoplasm |
| Proteasome subunit alpha type-1 | P25786 | 29.6 | 1 | 5.7 | 2 | Cytoplasm |
| Proteasome subunit beta type-2 | P49721 | 22.8 | 1 | 9.5 | 2 | Cytoplasm |
| Protein disulfide-isomerase A3 | P30101 | 56.8 | 1 | 2.2 | 2 | Endoplasmic reticulum |
| Protein DJ-1 | Q99497 | 19.9 | 1 | 7.9 | 2 | Cytoplasm |
| Protein S100-A9 | P06702 | 13.2 | 2 | 13.2 | 4 | Cytoplasm |
| Putative Rab-43-like protein ENSP00000330714 | A6NDJ8 | 20.2 | 1 | 6.1 | 2 | Cytoplasm |
| Putative Ras-related protein Rab-1C | Q92928 | 22.0 | 1 | 5.5 | 2 | Cytoplasm |
| Radixin | P35241 | 68.6 | 1 | 7.7 | 2.12 | Plasma membrane |
| Ras and EF-hand domain-containing protein | Q8IZ41 | 82.9 | 1 | 1.8 | 2 | Nucleus |
| Ras-related protein Rab-10 | P61026 | 22.5 | 1 | 5.5 | 2 | Cytoplasm |
| Ras-related protein Rab-12 | Q6IQ22 | 27.2 | 1 | 4.5 | 2 | Cytoplasm |
| Ras-related protein Rab-14 | P61106 | 23.9 | 1 | 5.1 | 2 | Cytoplasm |
| Ras-related protein Rab-15 | P59190 | 24.4 | 1 | 5.2 | 2 | Cytoplasm |
| Ras-related protein Rab-1A | P62820 | 22.7 | 1 | 5.4 | 2 | Cytoplasm |
| Ras-related protein Rab-1B | Q9H0U4 | 22.2 | 1 | 5.5 | 2 | Cytoplasm |
| Ras-related protein Rab-27A | P51159 | 24.9 | 1 | 5.9 | 2 | Cytoplasm |
| Ras-related protein Rab-27B | O00194 | 24.6 | 1 | 6.0 | 2 | Cytoplasm |
| Ras-related protein Rab-30 | Q15771 | 23.1 | 1 | 5.4 | 2 | Cytoplasm/cytoskeleton |
| Ras-related protein Rab-33B | Q9H082 | 25.7 | 1 | 4.8 | 2 | Cytoplasm |
| Ras-related protein Rab-34 | Q9BZG1 | 29.0 | 1 | 7.3 | 2 | Cytoplasm |
| Ras-related protein Rab-35 | Q15286 | 23.0 | 1 | 24.4 | 2 | Cytoplasm |
| Ras-related protein Rab-37 | Q96AX2 | 24.8 | 1 | 8.5 | 2 | Cytoplasm |
| Ras-related protein Rab-39A | Q14964 | 25.0 | 1 | 6.0 | 2 | Cytoplasm |
| Ras-related protein Rab-39B | Q96DA2 | 24.6 | 1 | 5.2 | 2 | Cytoplasm |
| Ras-related protein Rab-3A | P20336 | 25.0 | 1 | 5.0 | 2 | Cytoplasm/cytoskeleton |
| Ras-related protein Rab-3B | P20337 | 24.8 | 1 | 5.0 | 2 | Cytoplasm |
| Ras-related protein Rab-3C | Q96E17 | 26.0 | 1 | 4.8 | 2 | Cytoplasm |
| Ras-related protein Rab-3D | O95716 | 24.3 | 1 | 12.3 | 2 | Cytoplasm |
| Ras-related protein Rab-41 | Q5JT25 | 25.0 | 1 | 5.0 | 2 | Cytoplasm |
| Ras-related protein Rab-43 | Q86YS6 | 23.3 | 1 | 5.2 | 2 | Cytoplasm |
| Ras-related protein Rab-44 | Q7Z6P3 | 77.6 | 1 | 6.8 | 2.14 | Plasma membrane |
| Ras-related protein Rab-4A | P20338 | 24.4 | 1 | 5.0 | 2 | Mitochondrion |
| Ras-related protein Rab-4B | P61018 | 23.6 | 1 | 5.2 | 2 | Mitochondrion |
| Ras-related protein Rab-6A | P20340 | 23.6 | 1 | 6.3 | 2 | Cytoplasm |
| Ras-related protein Rab-6B | Q9NRW1 | 23.5 | 1 | 6.3 | 2 | Cytoplasm |
| Ras-related protein Rab-8A | P61006 | 23.7 | 1 | 5.3 | 2 | Cytoplasm |
| Ras-related protein Rab-8B | Q92930 | 23.6 | 1 | 5.3 | 2 | Cytoplasm |
| Ras-related protein Rab-9B | Q9NP90 | 22.7 | 1 | 5.5 | 2 | Extracellular |
| Serotransferrin | P02787 | 77.1 | 6 | 15.3 | 13.03 | Extracellular |
| Serum albumin5 | P02768 | 69.4 | 37 | 54.8 | 62.75 | Extracellular |
| Serum amyloid P-component | P02743 | 25.4 | 3 | 17.0 | 6 | Extracellular |
| SH3 domain-binding glutamic acid-rich-like protein | O75368 | 12.8 | 1 | 8.8 | 2 | Mitochondrion |
| Superoxide dismutase [Cu-Zn] | P00441 | 15.9 | 3 | 6.5 | 4 | Cytoplasm |
| Superoxide dismutase [Mn], mitochondrial | P04179 | 24.7 | 1 | 6.3 | 2 | Mitochondrion |
| Sushi, von Willebrand factor type A, EGF and pentraxin domain-containing protein 1 | Q4LDE5 | 390.2 | 1 | 0.8 | 1.52 | Extracellular |
| Transgelin4 | Q01995 | 22.6 | 12 | 56.2 | 20.02 | Cytoplasm |
| Translocon-associated protein subunit delta | P51571 | 19.0 | 1 | 6.4 | 1.52 | Endoplasmic reticulum |
| Transthyretin | P02766 | 15.9 | 2 | 29.9 | 3.7 | Extracellular |
| Triosephosphate isomerase | P60174 | 30.8 | 3 | 25.5 | 5.72 | Cytoplasm |
| Tripeptidyl-peptidase 1 | O14773 | 61.2 | 2 | 13.9 | 4.01 | Extracellular |
| Tropomyosin alpha-1 chain5 | P09493 | 32.7 | 1 | 3.5 | 2 | Cytoplasm |
| Tropomyosin alpha-3 chain5 | P06753 | 33.0 | 1 | 3.5 | 2 | Cytoplasm |
| Tropomyosin alpha-4 chain5 | P67936 | 28.5 | 1 | 4.0 | 2 | Cytoplasm |
| Tropomyosin beta chain5 | P07951 | 32.9 | 1 | 3.5 | 2 | Cytoplasm |
| Tryptase alpha/beta-1 | Q15661 | 30.5 | 4 | 18.5 | 8.47 | Extracellular |
| Tryptase beta-2 | P20231 | 30.5 | 4 | 18.5 | 8.47 | Extracellular |
| Tryptase delta | Q9BZJ3 | 26.6 | 1 | 13.6 | 3.05 | Extracellular |
| Tubulin alpha-1A chain5 | Q71U36 | 50.1 | 1 | 12.6 | 2 | Cytoplasm/cytoskeleton |
| Tubulin alpha-1B chain5 | P68363 | 50.2 | 1 | 12.6 | 2 | Cytoplasm/cytoskeleton |
| Tubulin alpha-1C chain5 | Q9BQE3 | 49.9 | 1 | 8.9 | 2 | Cytoplasm/cytoskeleton |
| Tubulin beta chain4 | P07437 | 49.7 | 3 | 11.5 | 6.03 | Cytoplasm/nucleus |
| Tubulin beta-2A chain | Q13885 | 49.9 | 2 | 9.4 | 4.03 | Cytoplasm |
| Tubulin beta-2B chain | Q9BVA1 | 50.0 | 2 | 9.4 | 4.03 | Cytoplasm |
| Tubulin beta-3 chain | Q13509 | 50.4 | 1 | 9.8 | 2.01 | Cytoplasm/cytoskeleton |
| Tubulin beta-4A chain | P04350 | 49.6 | 2 | 7.4 | 4.03 | Cytoplasm/nucleus |
| Tubulin beta-4B chain | P68371 | 49.8 | 3 | 11.5 | 6.03 | Cytoplasm/nucleus |
| Tubulointerstitial nephritis antigen-like | Q9GZM7 | 52.4 | 1 | 1.9 | 2 | Extracellular |
| Tumor-associated calcium signal transducer 2 | P09758 | 35.7 | 1 | 7.4 | 2 | Plasma membrane |
| Ubiquitin-40S ribosomal protein S27a5 | P62979 | 18.0 | 1 | 8.3 | 2 | Cytoplasm/nucleus |
| Ubiquitin-60S ribosomal protein L405 | P62987 | 14.7 | 1 | 10.2 | 2 | Extracellular |
| Vitronectin | P04004 | 54.3 | 2 | 15.1 | 4.03 | Extracellular |
| Zinc-alpha-2-glycoprotein | P25311 | 34.3 | 4 | 28.5 | 8.85 | Extracellular |

1. Also identified by 2DE-MALDI. [↑](#endnote-ref-1)
2. Also identified by Gel-MS/MS. [↑](#endnote-ref-2)
