# Supplementary material for: Proteins from formalin-fixed paraffin-embedded prostate cancer sections that predict the risk of metastatic disease
Source: Clin Proteomics. 2015 Sep 16;12(1):24. doi: 10.1186/s12014-015-9096-3 (PMC4574128; doi:10.1186/s12014-015-9096-3)
Supplement: Supplementary file 6 — Additional file 6: Summary of the proteins identified in FFPE PCa tissue that were selected for Western blot (WB) analysis. [file 12014_2015_9096_MOESM6_ESM.docx]

**Additional file 6. Summary of the proteins identified in FFPE PCa tissue that were selected for Western blot (WB) analysis.**

| Protein | Gene ID | Uniprot ID | HPA candidate^[[1]](#footnote-1)^ | HPA staining PCa tissue | Selected for WB analysis^[[2]](#footnote-2)^ | Detectable by WB |
| --- | --- | --- | --- | --- | --- | --- |
| Annexin A2 | ANXA2 | P07355 | Yes | High | Yes | Yes |
| Calmodulin | CALM1 | P62158 | Yes | High | No | - |
| Cathepsin D | CTSD | P07339 | Yes | Medium | No | - |
| Galectin-3-binding protein | LGALS3BP | Q08380 | Yes | Medium | No | - |
| Growth/differentiation factor 15 | GDF15 | Q99988 | Yes | High | No | - |
| Heat shock 70 kDa protein 1A | HSPA1A | P08107 | Yes | Medium | Yes | No |
| Heat shock protein beta-1 | HSPB1 | P04792 | Yes | Medium | Yes | Yes |
| Heat shock-related 70 kDa protein 2 | HSPA2 | P54652 | Yes | High | No | - |
| Macrophage migration inhibitory factor | MIF | P14174 | Yes | High | No | - |
| Prostate-specific antigen | KLK3 | P07288 | Yes | High | Yes | Yes |
| Prostatic acid phosphatase | ACPP | P15309 | Yes | High | No | - |
| Zinc-alpha-2-glycoprotein | AZGP1 | P25311 | Yes | High | Yes | Yes |
| Peroxiredoxin-1 | PRDX1 | Q06830 | No | Medium | Yes | Yes |
| Protein DJ-1 | PARK7 | Q99497 | No | High | Yes | No |

Selection of candidates for Western blotting.

Candidates for Western blotting were selected from the set of proteins that had been identified by MS (Additional information 3-5) using a decision making process based on high quality antibody reactivity data mined from the Human Protein Atlas (HPA, <http://www.proteinatlas.org/>).

The HPA was searched for antibody reactivity using the following fields: keyword “Prostate”; protein class “Candidate cancer biomarkers”; HPA evidence “High”^[[3]](#footnote-3)^, and “High/Medium” immunohistochemical reaction with prostate tumour tissue. The resulting set of 98 proteins included 12 of the 320 proteins that had been identified using 2DE, Gel-MS/MS or LC-MS/MS. Five of the 12 proteins that were at the interface of the HPA and MS lists were then selected as candidates for testing by Western blotting based on published information about their potential roles in PCa. The selected candidates were annexin A2, heat shock 70 kDa protein 1A, heat shock protein beta-1, prostate-specific antigen and zinc-alpha-2-glycoprotein; but heat shock 70 kDa protein 1A could not be detected by Western blotting. Peroxiredoxin-1, that was not found in the HPA list but was detected by MS, was also trialed because of known associations with PCa.

In summary, five (annexin A2, heat shock protein beta-1, peroxiredoxin-1, prostate-specific antigen and zinc-alpha-2-glycoprotein) of the seven tested candidates were detected readily using Western blotting of radical prostatectomy tissue, and were advanced for analysis of archival FFPE biopsies in the pilot study for prediction of risk of metastatic disease.

1. Protein selected from the HPA database using keywords “Prostate”, “Candidate cancer biomarkers”, “High” HPA evidence, and “High” or “Medium” IHC staining in normal prostate glandular tissue. [↑](#footnote-ref-1)
2. Published information suggesting that the protein has a role in PCa progression and/or metastasis, and therefore selected as a candidate for examination of Western blot detectability in radical prostatectomy PCa tissue.. [↑](#footnote-ref-2)
3. HPA evidence scores are based on the quality of antibody data including in Western blots and tissue profiling (Fagerberg L, Oksvold P, Skogs M, Algenäs C et *al*. Contribution of antibody-based protein profiling to the human Chromosome-centric Proteome Project (C-HPP). J Proteome Res. 2013, *12*, 2439-2448. [↑](#footnote-ref-3)
