# Supplementary material for: Proteins from formalin-fixed paraffin-embedded prostate cancer sections that predict the risk of metastatic disease
Source: Clin Proteomics. 2015 Sep 16;12(1):24. doi: 10.1186/s12014-015-9096-3 (PMC4574128; doi:10.1186/s12014-015-9096-3)

**Additional file 7. Biochemical failure free survival as a function of PSA risk stratification group.** Kaplan-Meier curves for PSA, using a priori selected three-fold cut-off for protein abundance in tumour relative to control tissue in each of the 16 cases, demonstrated a significant difference in failure risk (log-rank chi-squared p = 0. 016). All men with more than a three-fold PSA abundance increase experienced biochemical failure within 87 months of diagnosis. In contrast, more than 55% of men with less than a three-fold PSA increase remained biochemical failure free. Vertical lines on PSA < 3-fold change curve denote censored cases.

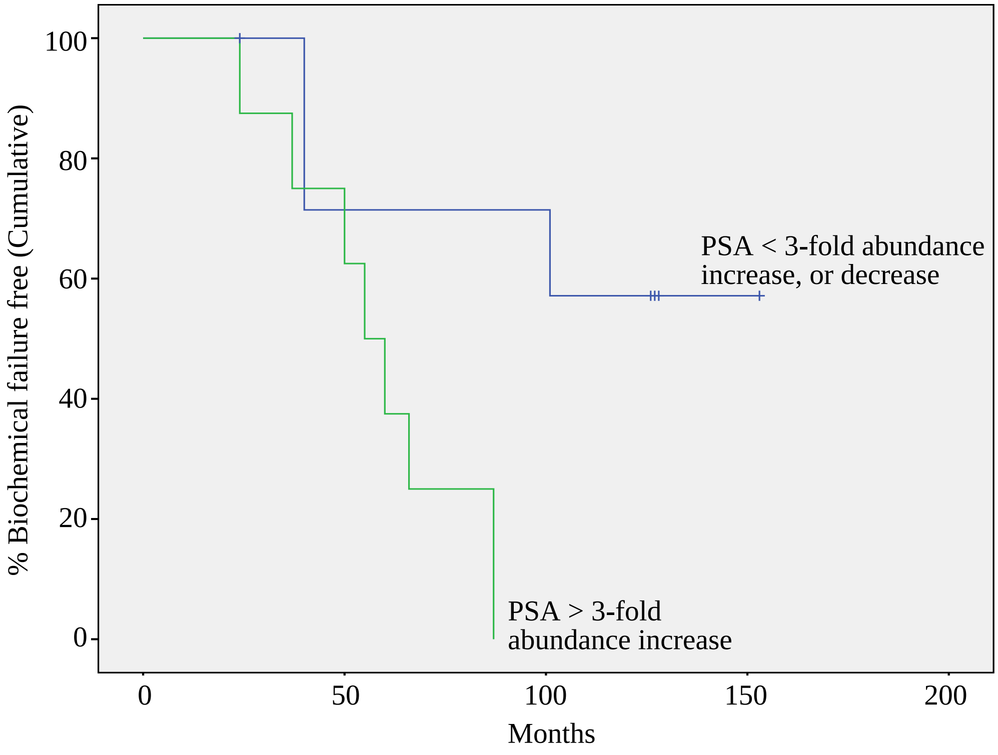

Supplement: Supplementary file 7 — Additional file 7: Biochemical failure free survival as a function of PSA risk stratification group. [file 12014_2015_9096_MOESM7_ESM.pdf]
