# Supplementary material for: Proteins from formalin-fixed paraffin-embedded prostate cancer sections that predict the risk of metastatic disease
Source: Clin Proteomics. 2015 Sep 16;12(1):24. doi: 10.1186/s12014-015-9096-3 (PMC4574128; doi:10.1186/s12014-015-9096-3)
Supplement: Supplementary file 8 — Additional file 8: Summary of patient samples analysed. [file 12014_2015_9096_MOESM8_ESM.docx]

**Additional file 8. Summary of patient samples analysed.**

| Treatment arm.  RT, radiotherapy; HT, hormone therapy. | Gleason score | Age at randomization (years) | Time to biochemical failure or censoring (months) |
| --- | --- | --- | --- |
| RT + 6mth HT | 3+3 | 55 | 127 |
| RT + 6mth HT | 3+3 | 77 | 37 |
| RT + 6mth HT | 3+3 | 58 | 24 |
| RT + 6mth HT | 3+3 | 61 | 24 |
| RT + 3mth HT | 3+3 | 75 | 128 |
| RT + 6mth HT | Specimen 5 & 6: Gleason 3 | 71 | 60 |
| RT + 6mth HT | 4+3 | 52 | 55 |
| RT + 6mth HT | 2+1 | 63 | 153 |
| RT + 3mth HT | 5+5 | 58 | 40 |
| RT + 3mth HT | 4+3 | 68 | 126 |
| RT + 6mth HT | Gleason primary grade 3 | 65 | 66 |
| RT + 3mth HT | Gleason grade 5 involving 20% grade 4 & 30% o grade 6 | 58 | 87 |
| RT + 3mth HT | 4+5 | 62 | 40 |
| RT + 3mth HT | 4+5 | 66 | 87 |
| RT + 6mth HT | 3+4 | 69 | 50 |
| RT + 3mth HT | 3+3 | 64 | 101 |
